# Early Detection of Malignant Pleural Mesothelioma in Asbestos-Exposed Individuals with a Noninvasive Proteomics-Based Surveillance Tool

**DOI:** 10.1371/journal.pone.0046091

**Published:** 2012-10-03

**Authors:** Rachel M. Ostroff, Michael R. Mehan, Alex Stewart, Deborah Ayers, Edward N. Brody, Stephen A. Williams, Stephen Levin, Brad Black, Michael Harbut, Michele Carbone, Chandra Goparaju, Harvey I. Pass

**Affiliations:** 1 Clinical Research, SomaLogic Inc., Boulder, Colorado, United States of America; 2 Center for Occupational and Environmental Medicine, Mt. Sinai Medical Center, New York, New York, United States of America; 3 Center for Asbestos Related Diseases, Libby, Montana, United States of America; 4 Environmental Cancer Program, Karmanos Cancer Center, Detroit, Michigan, United States of America; 5 Department of Pathology, Cancer Research Center of Hawaii, Honolulu, Hawaii, United States of America; 6 Department of Cardiothoracic Surgery, New York University Langone Medical Center and Cancer Center, New York, New York, United States of America; University of Bochum, Germany

## Abstract

**Background:**

Malignant pleural mesothelioma (MM) is an aggressive, asbestos-related pulmonary cancer that is increasing in incidence. Because diagnosis is difficult and the disease is relatively rare, most patients present at a clinically advanced stage where possibility of cure is minimal. To improve surveillance and detection of MM in the high-risk population, we completed a series of clinical studies to develop a noninvasive test for early detection.

**Methodology/Principal Findings:**

We conducted multi-center case-control studies in serum from 117 MM cases and 142 asbestos-exposed control individuals. Biomarker discovery, verification, and validation were performed using SOMAmer proteomic technology, which simultaneously measures over 1000 proteins in unfractionated biologic samples. Using univariate and multivariate approaches we discovered 64 candidate protein biomarkers and derived a 13-marker random forest classifier with an AUC of 0.99±0.01 in training, 0.98±0.04 in independent blinded verification and 0.95±0.04 in blinded validation studies. Sensitivity and specificity at our pre-specified decision threshold were 97%/92% in training and 90%/95% in blinded verification. This classifier accuracy was maintained in a second blinded validation set with a sensitivity/specificity of 90%/89% and combined accuracy of 92%. Sensitivity correlated with pathologic stage; 77% of Stage I, 93% of Stage II, 96% of Stage III and 96% of Stage IV cases were detected. An alternative decision threshold in the validation study yielding 98% specificity would still detect 60% of MM cases. In a paired sample set the classifier AUC of 0.99 and 91%/94% sensitivity/specificity was superior to that of mesothelin with an AUC of 0.82 and 66%/88% sensitivity/specificity. The candidate biomarker panel consists of both inflammatory and proliferative proteins, processes strongly associated with asbestos-induced malignancy.

**Significance:**

The SOMAmer biomarker panel discovered and validated in these studies provides a solid foundation for surveillance and diagnosis of MM in those at highest risk for this disease.

## Introduction

Malignant mesothelioma (MM) is a relatively rare cancer almost always caused by prolonged exposure to asbestos fibers. There are about 2,500–3,000 new cases per year in the USA [1]. Although the disease is not frequent, it is devastating, with a median survival of 7 months [2]. Furthermore, over 27 million people in the US, and millions more worldwide, have been exposed to asbestos fibers and thus are at risk for the disease. There are 15,000–20,000 deaths per year from MM in the Western world and Japan [2]. Since the most exposed, and therefore most at risk, people have been exposed through their occupations (miners, pipe-coverers, shipyard workers, etc.), there are important medico-legal issues involved almost every time a person is diagnosed with MM [1].

Definitive diagnosis of MM requires distinguishing it from benign pleural disease, such as asbestosis or other inflammatory conditions, or from metastasis of other primary cancers to the pleura. Diagnosis is difficult and depends on invasive sampling of pleural fluid or tissue. Currently the most prescribed screening methods for surveillance of asbestos-exposed patients involve imaging procedures that are costly and expose patients to high doses of radiation each year. In addition, the high rate of incidental imaging findings requiring follow up for nonmalignant conditions leads to unnecessary invasive procedures, patient anxiety and cost [3], [4].

Blood-based biomarkers for differential diagnosis and monitoring treatment response of MM include mesothelin and its proteolytic products and osteopontin [5], [6]. Mesothelin is reported to have low sensitivity (32%) for early disease [6]–[8], but early detection may be improved with serial sampling in a high-risk population [9]. Osteopontin has shown promise for early detection, but serum protein instability has led to variable results [2]. More recently, changes in micro-RNAs have been reported in tissue and blood to have diagnostic potential when combined with mesothelin [10] or as prognostic markers correlated with progression and overall survival [11].

Since MM is a low incidence disease even in the asbestos-exposed population, a need still exists for a highly specific test for risk surveillance and early detection while avoiding false positive results and unnecessary invasive procedures. We report the discovery and validation of a serum-based 13-protein classifier with an AUC of 0.95 and an overall accuracy of 92% for detection of MM in the asbestos-exposed population using the SOMAscan™ proteomic assay. This assay utilizes Slow Off-rate Modified Aptamers (SOMAmers™) to quantify proteins in biologic samples [12]. SOMAmers are selected to have slow specific off-rates for dissociation of targeted analytes, which results in highly selective protein detection [13]. The biomarker discovery assay measures more than 1000 proteins in biologic samples without sample depletion or fractionation. Once biomarkers have been identified, targeted panels for specific diagnostic applications can be assembled from the same SOMAmers, thus simplifying the transition for discovery to clinical use [14].

The use of SOMAmers as capture reagents offers several advantages over traditional antibodies [14]. The synthetic nature of SOMAmers ensures uniformity and consistent availability. Customization of the affinity reagent with chemical attachment or signaling moieties is routine, relying only on the availability of the appropriate phosphoramidites. SOMAmers have the chemical and thermal stability properties of DNA, which exceeds that of proteins, including antibodies. SOMAmers typically bind to large structural portions of their protein target and therefore require the protein to be properly folded for optimal recognition [12], [13], making consistent sample processing an essential requirement for accurate measurement.

The use of SOMAmers as capture reagents carries advantages over traditional antibody-based arrays. The intrinsic upper limit of high sensitivity antibody arrays to multiplexing 30–40 analytes is not a constraint with SOMAmer arrays, which currently measure over 1000 proteins. Sensitive antibody arrays require two antibodies per analyte to avoid cross-reactivity, but the slow-off rate selection of SOMAmers provides specificity in binding with only a single SOMAmer per protein target [13].

## Materials and Methods

### Objectives

The objective of this study was to apply the SOMAscan proteomic assay to discover and validate serum-based biomarkers for detection of MM in the asbestos-exposed, at risk population.

### Participants

Serum samples from MM cases and asbestos-exposed controls were collected at 4 institutions: New York University (NYU), Mount Sinai Medical Center (SIN), the Center for Asbestos Related Diseases in Libby, Montana (LIB) and Karmanos Cancer Institute (KAR) (Tables 1 and 2). The MM cases were consecutively collected in the clinics (pre-op) or at the time of surgery (intra-op) at KAR (1996–2005) and NYU (2005–2011). Additional serum from 6 benign and 26 malignant (non-MM) pleural effusion subjects was obtained from NYU. All MM cases were pathologically confirmed by cytology and/or resection by a specialist in mesothelioma pathology (co-author MC), and consenting patients were eligible for inclusion in the study whether they had symptoms or not. Blood samples were collected from most cases prior to treatment. Control blood was obtained from study participants with a history of asbestos exposure. The control group contains individuals with asbestosis, pulmonary fibrosis and pulmonary plaques and represents the population most at risk for MM. The KAR asbestos-exposed cohort were patients followed at the Center for Occupational and Environmental Medicine (co-author MH) who consented for study participation between 2003 and 2005, and included foundry workers, pipe fitters, building and construction, passive exposure from construction or a family member, brake assembly or repair, boiler repair, vermiculite exposure, plumbers, ship builders, machinists, tool and die workers, millwrights, brick layers, and electricians [6]. Radiographic evidence of fibrosis was found in 33%, and pleural scarring/plaques were found in 75%. The SIN asbestos exposed cohort included active and retired insulators enrolled in a follow-up to the Selikoff Cohort program [15] with 63% having pleural scarring, 24% with plaques, and 5% having parenchymal changes. The LIB asbestos-exposed cohort included individuals who were seen at the Center for Asbestos Related Diseases between 2004 and 2006 who were involved with the mining or processing of tremolite contaminated vermiculite and who had pleural changes on computerized tomography.

**Table 1:** Study cohort (n = 259) by blood collection site.

Site	Cases (n = 117)	Controls (n = 142)	Total/Site
**NYU**	66	0	66
**KAR**	51	41	92
**LIB**	0	71	71
**SIN**	0	30	30

**Table 2:** Cohort demographics.

		Training (n = 120)		Verification (n = 39)		Validation (n = 100)	
		Case	Control	Case	Control	Case	Control
**Number**		60	60	19	20	38	62
**Gender (%)**	**Male**	50 (83)	41 (68)	17 (89)	16 (80)	31 (82)	43 (69)
	**Female**	10 (17)	19 (32)	2 (11)	4 (20)	7 (18)	19 (31)
**Age**	**Median**	64	62	64	66	64	62
	**Range**	41–91	36–90	50–79	42–80	41–87	22–80
**Asbestos Exp (%)**		45 (75)	55 (92)	14 (74)	18 (90)	25 (66)	61 (98)
**MM Stage (%)**	**I**	7 (12)	NA	1 (5)	NA	5 (13)	NA
	**II**	10 (17)	NA	4 (21)	NA	13 (34)	NA
	**III**	30 (50)	NA	9 (47)	NA	10 (26)	NA
	**IV**	13 (22)	NA	4 (21)	NA	9 (24)	NA
	**Unknown**	0 (0)	NA	1 (5)	NA	1 (3)	NA
**MM Histology (%)**	**Epithelial**	39 (65)	NA	9 (47)	NA	16 (42)	NA
	**Biphasic**	12 (20)	NA	8 (42)	NA	6 (16)	NA
	**Sarcomatoid**	4 (7)	NA	1 (5)	NA	3 (8)	NA
	**Unknown**	5 (8)	NA	1 (5)	NA	13 (34)	NA

### Ethics

All samples and clinical information were collected under Health Insurance Portability and Accountability Act (HIPAA) compliance from study participants after obtaining written informed consent under clinical research protocols approved by the institutional review boards for each site. The NYU Langone Medical Center Institution Review Board approved this study. Demographic data was collected by self-report and clinical data by chart review.

### Sample Collection Procedure

Serum samples were collected following uniform processing protocols recommended by the National Cancer Institute's Early Detection Research Network (EDRN) using red top Vacutainer tubes (Becton Dickinson, Raritan, NJ) [16]. Processing time from blood collection to centrifugation was 1–6 hours. All samples were stored at −80°C. Samples were collected either intra-op or pre-op from MM cases and during routine clinic visits for asbestos-exposed controls. To control for biomarker differences resulting from the blood draw procedure, paired intra-op and pre-op blood samples were compared from the same individuals. Any candidate biomarkers affected by the blood draw procedure were removed from the analysis.

### Sample Blinding

To prevent potential bias, a unique unidentifiable barcode was assigned to each sample and data record, and the key was stored in a secure database accessible only to designated study administrators. The sample blinding code was broken according to the pre-specified analysis plan. First a subset was unmasked for training the classifier. Unmasking the samples for classifier verification and validation occurred only after the classifier was fixed. For the verification sample set, a blinding key was provided exclusively to a third party reader, unaffiliated with the study centers or SomaLogic, for calculating final results.

### Proteomic Analysis

Serum samples (15 µl) were analyzed on the SOMAscan proteomic assay, which uses novel modified DNA aptamers called SOMAmers to specifically bind protein targets in biologic samples [12], [13]. All sample analyses were conducted in the Good Laboratory Practice (GLP) compliant lab at SomaLogic by trained staff. Serum samples were distributed randomly in 96-well microtiter plates and the assay operators were blinded to case/control identity of all samples. Assay results are reported in Relative Fluorescence Units (RFU). Data processing was as described by Gold [12]. Briefly, microarray images were captured and processed with a microarray scanner and associated software. Each sample in a study was normalized by aligning the median of each sample to a common reference. Inter-plate calibration was done by applying a multiplicative scaling coefficient to each SOMAmer. These scaling factors were calculated using the eight reference calibrators on each plate.

The biomarker discovery and verification studies were conducted with Version 1 (V1) of the assay, which measured over 800 proteins [12]. The final validation study used Version 2 (V2), which measures 1045 proteins (Table S1). Minor assay protocol changes were incorporated in V2 to optimize the sample diluent and washing steps. The classifier containing the same 13 candidate biomarkers was re-trained in the V2 format with a bridging study which included 113 of the original 120 training samples; 7 samples were depleted after the initial training. Equivalent performance was demonstrated with a Spearman correlation coefficient of 0.92 prior to blinded verification and validation (Figure S1).

### Candidate Biomarker Selection and Classifier Training

**Figure 1:**
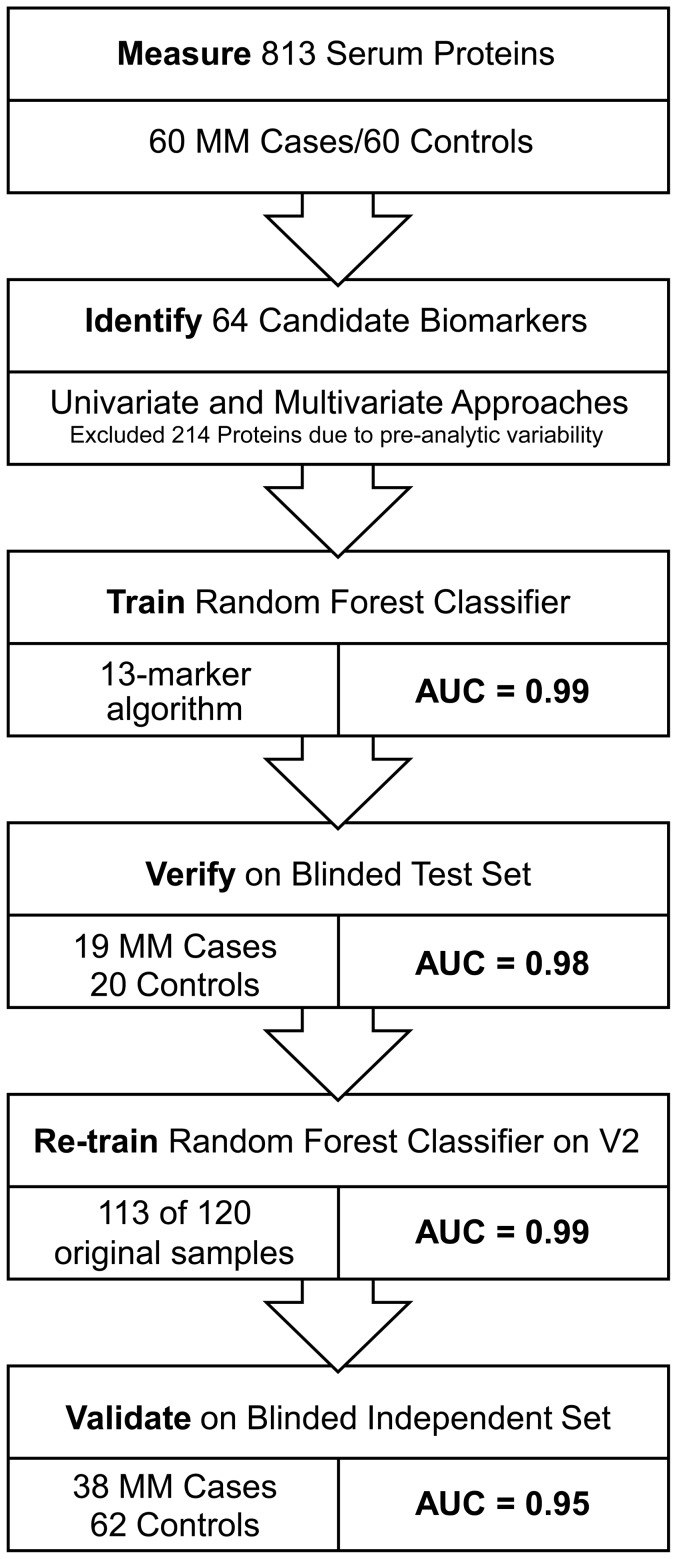
Study flowchart for classifier training, blinded verification and validation. Biomarker selection and training were performed with V1 of the assay. The equivalent classifier was applied to verification and validation studies using V2 of the assay.

The cohort of 159 samples was divided randomly into two sets, 75% for training (60 cases/60 controls) and cross-validation and 25% (19 cases/20 controls) for blinded verification, which were withheld from training to test classifier performance (Figure 1). This was followed by a blinded independent validation set of 100 samples (38 cases/62 controls). A series of univariate and multivariate comparisons were made to identify candidate MM biomarkers and filter out analytes subject to preanalytical variability. A 13 biomarker random forest classifier was applied to the blinded verification and validation study samples to predict the probability of MM. Functional analysis was performed with DAVID Bioinformatics Resources version 6.7 [17].

### Statistical Analysis for Candidate Biomarker Selection and Classifier Construction

A major issue with diagnostic discovery, particularly when using archived sample sets is the possibility that systematic batch effects may distort the results and lead to errors in the selection of candidate disease biomarkers. The development of the diagnostic panel presented here was performed on a large data set with samples from multiple sites, which was designed to detect variations in sample preparation and to allow us to mitigate the risks associated with preanalytic variability. Of particular importance was the collection of paired pre-op and intra-op samples from twelve control individuals, because many of our MM samples were drawn intra-op, whereas most control samples were standard clinic draws. Having a wide range of control sample sets allowed us to exclude 214 potential markers which showed variation between the different control sample sets (Kolmogorov-Smirnov (KS) distance >0.45), or between matched intra-op and pre-op samples. Principal Components Analysis (PCA) was used to exclude samples and analytes that showed evidence of bias due to preanalytic variation. Samples and analytes with high coefficients on principal components associated with different sources of preanalytic variability were removed. The principal components associated with preanalytic variation were identified by correlating them with previous clinical experiments on preanalytic variation in blood sample collection [18]. As a result, one set of 30 SIN control samples from asbestos exposed individuals was removed, as the samples were found to have suffered extensive protein degradation. These samples were not included in the cohort description (Tables 1 and 2).

After excluding the proteins shown to be susceptible to variation between control groups, we performed candidate marker selection on a training dataset composed of MM samples and the asbestos-exposed control samples. Candidate biomarkers were ranked used the random forest Gini importance measure, which reflects the magnitude of an individual marker's contribution to the classifier performance, calculated from the construction of a random forest classifier on the 64 candidate biomarkers [19]. We ranked the candidate markers by their Gini importance and compared the performance of various size models constructed using the highest ranked markers. Thirteen proteins were used to construct a random forest classifier on the data set. Ranking the candidate biomarkers once based on a single random forest model built using all biomarkers was chosen over stepwise selection/backwards elimination techniques to avoid complexity. Since the random forest importance measure is calculated on the out of bag samples, this approach to ranking candidate markers by a single application of random forest classification should be somewhat resistant to over-fitting. Other methods of marker selection (modified t-tests, KS tests), came up with similar lists of markers, with slightly different orderings.

The study design and execution were conducted according to accepted best practices [20]. Analyses were performed with R statistical software version 2.10.1. We used the R packages random forest (4.5–34) and fdrtool (1.2.6).

### ELISA Correlation Studies

Mesothelin was measured with the Mesomark Assay (Fujirebio Diagnostics) [5] and compared to SOMAscan results for 32 cases and 34 controls, using a cutoff of 1.9 nM as described. FCN-2 was measured in serum samples with the Human L-ficolin ELISA kit (Hycult biotech, Uden, the Netherlands). Complement Factor 9 (MicroVue SC5b-9 EIA kit, Quidel Corporation, San Diego, CA USA), Factor IX (AssayMax Human Factor IX ELISA kit, AssayPro St. Charles, MO USA) and Human CXCL13 (Human CXCL13/BLC/BCA-1 Quantikine ELISA kit, R&D Systems, Minneapolis, MN USA) were analyzed in order to validate SOMAmer results in the 68 controls and 32 MMs in the blinded validation trial.

## Results

We analyzed a total of 259 serum samples from four independent MM biorepositories in a series of prospectively designed case/control studies with archived samples (Figure 1 and Table 1). The study included serum collected from 117 MM patients and 142 high-risk controls, 94% of whom had documented asbestos exposure (Table 2). The remaining 6% of controls were individuals who had unusual occupations and included engineers who were not on site at high risk jobs, teachers, and nuclear power plant workers. They still participated in screening because of their association with others at high risk. One third of the MM cases had stage I or II disease, which enabled discovery of potential biomarkers of early disease and the possibility to identify patients with a chance for curative intervention.

**Figure 2:**
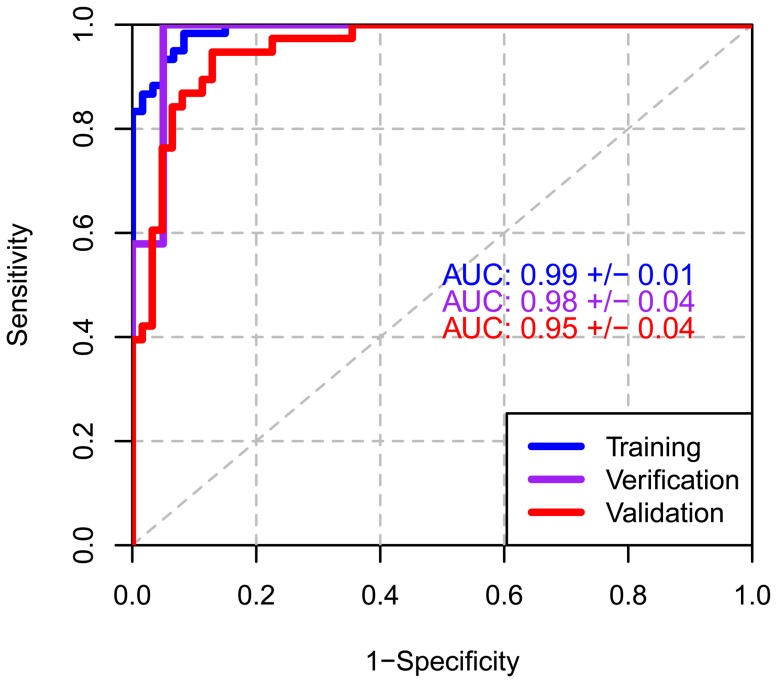
ROC curves for classifier training, blinded verification and validation. Training (blue), verification (purple), and validation (red) study ROC curves are plotted with corresponding AUC values and 95% confidence intervals.

Analysis of the training study yielded a set of 64 unique biomarker candidates (Table S1). We constructed a 13-protein random forest classifier from these potential biomarkers with an AUC of 0.99±0.01 in training and 0.98±0.04 in blinded verification (Figure 2). Sensitivity and specificity were 97%/92% in training and 90%/95% in blinded verification (Table 3). This classifier accuracy was maintained in the independent blinded validation set with an AUC of 0.95±0.04, and a sensitivity/specificity of 90%/89%. The combined sensitivity/specificity for all samples was 94%/91% resulting in an accuracy of 92% (Figure 2 and Table 3). Sensitivity correlated with pathologic stage (Table 4). Overall 77% of Stage I, 93% of Stage II, 96% of Stage III and 96% of Stage IV cases were detected. The sensitivity for detection of local disease (Stages I and II) was 88%, demonstrating that the classifier can identify the majority of MM at potentially curable stages with a higher chance for successful multimodality therapy. We also tested 32 individuals with non-MM pleural effusion (PE) and 30 asbestos-exposed controls. All 6 benign and 24/26 malignant PE samples were classified as disease.

**Table 3:** Classifier performance for training, verification and validation.

Study Set	Sensitivity	Specificity	Accuracy
**Training**	96.7 (92.1–100.0)	91.7 (84.7–98.7)	94.2 (90.0–98.4)
**Verification**	89.5 (75.7–100.0)	95.0 (85.4–100.0)	92.3 (83.9–100.0)
**Validation**	89.5 (79.7–99.2)	88.7 (80.8–96.6)	89.0 (82.9–95.1)
**Combined**	93.2 (88.6–97.7)	90.8 (86.1–95.6)	91.9 (88.6–95.2)

Percentage values at the predefined decision threshold and 95% confidence intervals.

**Table 4:** MM detection by pathologic stage and study cohort.

MM Stage	Train	Verification	Validation	Combined
**I**	6/7	0/1	4/5	10/13
**II**	10/10	4/4	11/13	25/27
**III**	29/30	8/9	10/10	47/49
**IV**	13/13	4/4	8/9	25/26
**Unknown**	0/0	1/1	1/1	2/2

**Figure 3:**
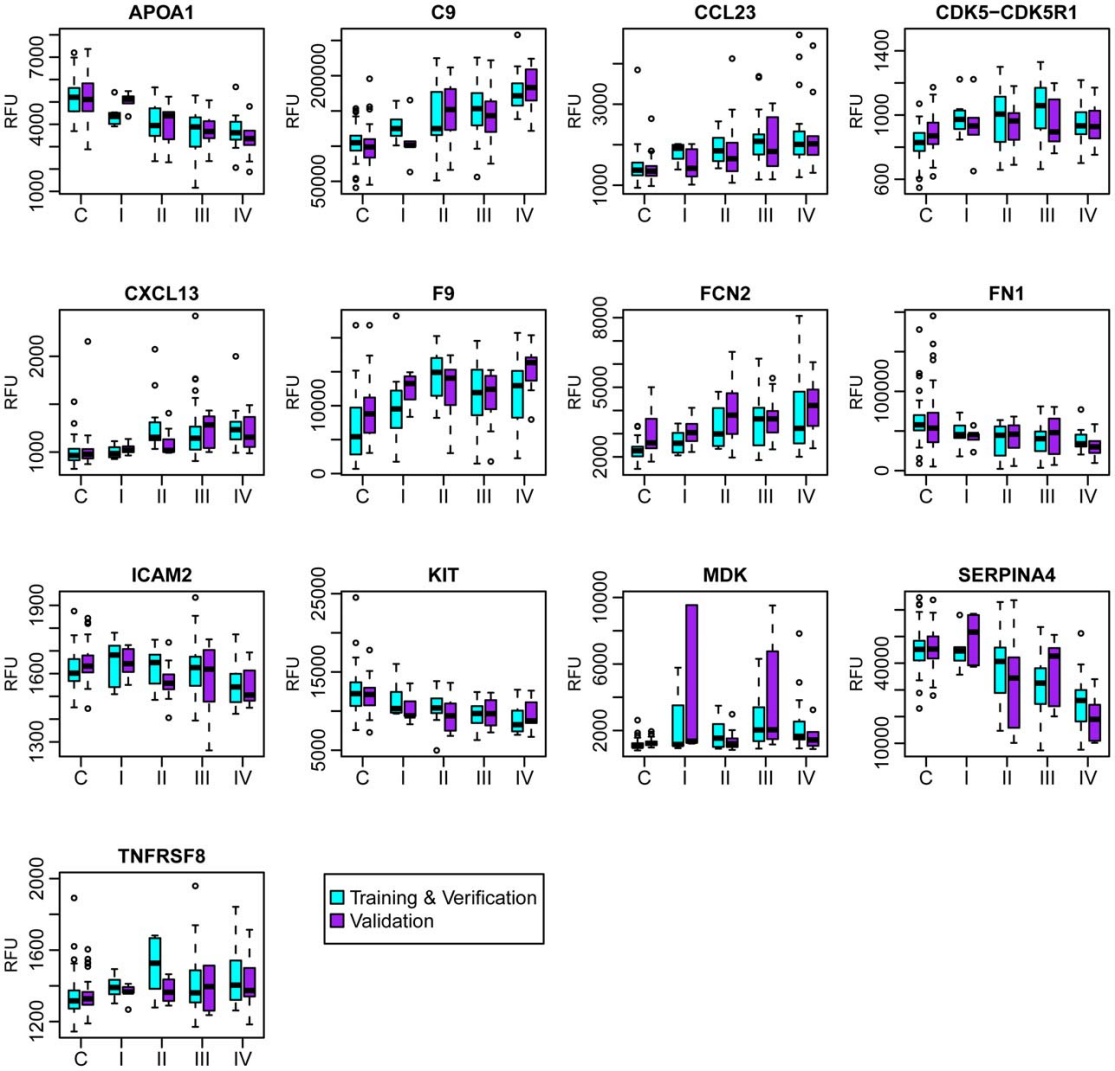
Distribution of the 13 protein biomarkers by pathologic stage. Teal boxes are samples from training and verification combined. Purple boxes are samples from the validation study. Relative fluorescence unit (RFU) distributions are separately shown for control (C) and pathologic stages (I–IV) to illustrate the change in signal as a function of disease burden. Some outlying points have been omitted to make the box plots easier to see: APOA1 (1 point), CDK5-CDK5R1 (1 point), MDK (6 points), and TNFRSF8 (8 points).

Table 5 lists the 13 candidate biomarkers along with their statistical significance for distinguishing MM from controls. Nine of the biomarkers are elevated in MM and 4 are lower compared to the asbestos-exposed controls. The measured protein values consistently reflect pathologic stage and disease burden (Figure 3). The ability of the classifier to detect MM was not compromised by neoadjuvant chemotherapy prior to blood draw or by histology. Ten patients received neoadjuvant therapy, and eight of them were correctly identified as MM. Across the three study cohorts there were eight false negative cases: six epithelial, one biphasic, and one mixed, which reflects the distribution of these histological categories in the cohort as a whole.

**Table 5:** Biomarkers in random forest classifier and their statistical significance.

Gene Name	Gene ID	Protein Target	SwissProt ID	Function	MM vs Asbestos*	KS test p-value	t test p-value
**APOA1**	335	Apo A-I	P02647	Lipid transport	Down	2.99E-08	6.32E-11
**C9**	735	C9	P02748	Adaptive immune response	Up	6.47E-07	1.14E-07
**CCL23**	6368	Ck-b-8-1	P55773	Cellular ion homeostasis, inflammatory response	Up	2.81E-06	4.00E-08
**CDK5/CDK5R1**	1020/8851	CDK5/p35	Q00535/Q15078	Cell morphogenesis	Up	1.22E-06	8.64E-09
**CXCL13**	10563	BLC	O43927	Immune system development	Up	1.67E-09	6.31E-08
**F9**	2158	Coagulation Factor IX	P00740	Coagulation cascade	Up	2.46E-07	9.61E-09
**FCN2**	2220	FCN2	Q15485	Immune effector	Up	3.38E-09	6.09E-11
**FN1**	2335	Fibronectin	P02751	Cell morphogenesis	Down	9.23E-06	9.41E-06
**ICAM2**	3384	sICAM-2	P13598	Cell adhesion	Up	2.67E-05	1.75E-06
**KIT**	3815	SCF sR	P10721	Immune system development, receptor tyrosine kinase	Down	3.83E-06	1.14E-08
**MDK**	4192	Midkine	P21741	Regulation of cell division	Up	2.99E-08	8.54E-02
**SERPINA4**	5267	Kallistatin	P29622	Serine protease inhibitor	Down	2.05E-07	4.56E-07
**TNFRSF8**	943	CD30	P28908	Regulation of cytokines & cell proliferation	Up	8.02E-08	3.94E-06

* Up or down regulation in MM cases relative to controls.

**Figure 4:**
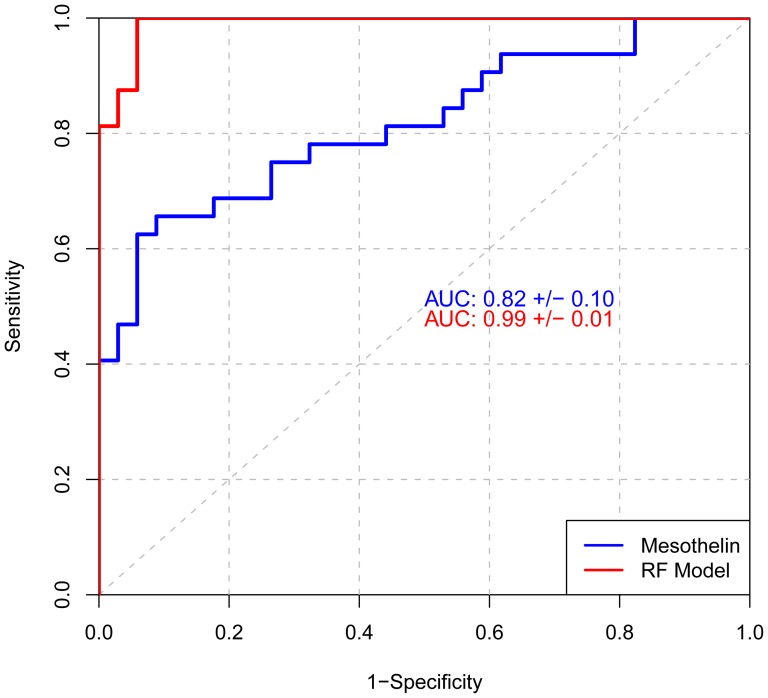
ROC curves comparing the random forest classifier to mesothelin. Performance of the random forest classifier (red) compared to a commercial mesothelin assay (blue) on the same cohort of 32 MM cases and 34 asbestos exposed controls. ROC curves are plotted with corresponding AUC values and 95% confidence intervals.

We compared the random forest classifier to mesothelin, as measured by a commercial ELISA. Applying the random forest classifier generated an AUC of 0.99+/−0.01 while the commercial ELISA for mesothelin resulted in an AUC of 0.82+/−0.10 (Figure 4). The sensitivity and specificity of mesothelin this cohort was 66%/88% compared to the random forest classifier sensitivity and specificity of 91%/94% in this paired sample set.

**Figure 5:**
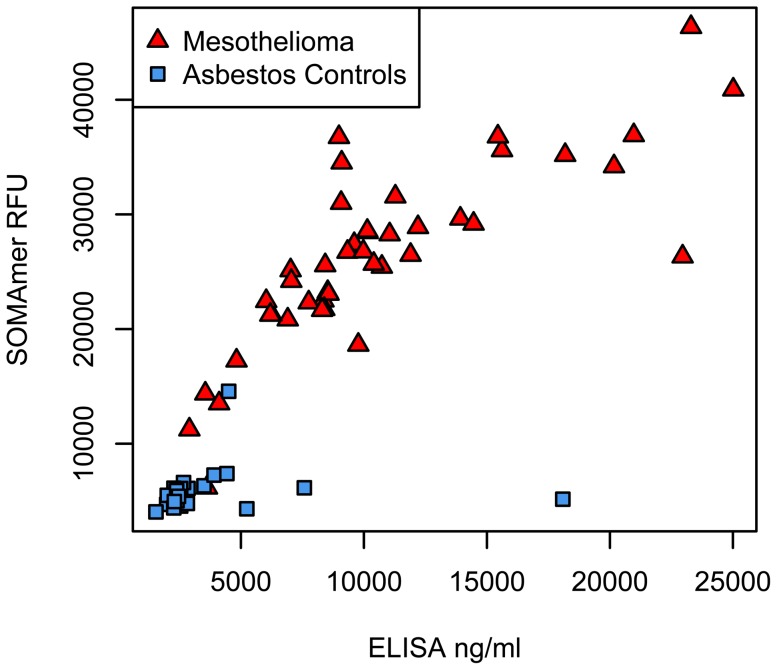
FCN2 SOMAmer and ELISA correlation in the training cohort. FCN2 measurements for MM cases (red triangles) and asbestos-exposed controls (blue squares) are reported as RFU for SOMAmer and ng/ml for ELISA measurements. Spearman correlation is 0.87.

**Figure 6:**
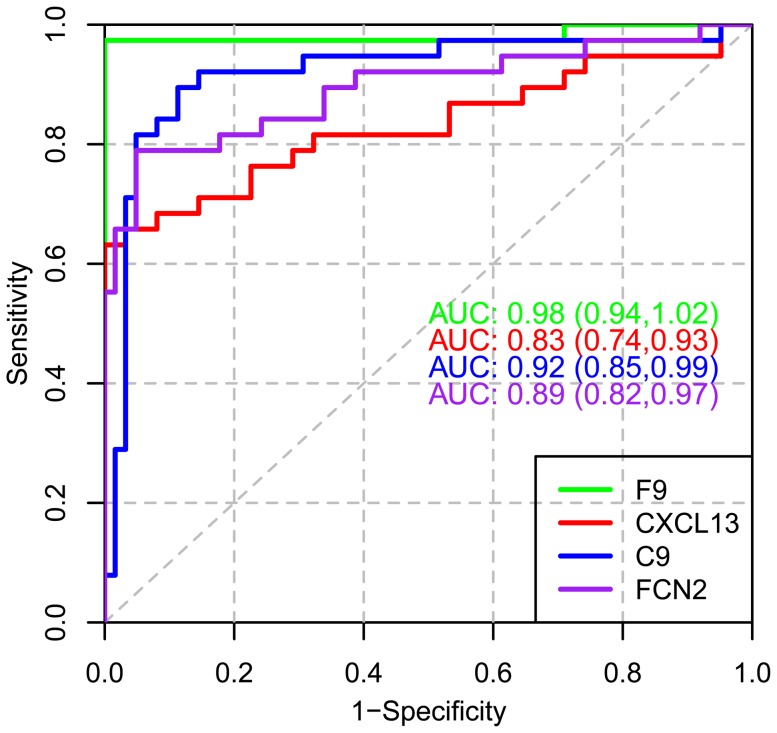
ROC curves of individual MM biomarkers measured by commercial ELISA kits. AUC values and 95% confidence intervals for F9 (green), CXCL13 (red), C9 (blue), FCN2 (purple) were derived from measurements in the validation study samples.

We compared the SOMAmer-measured values of one of the classifier proteins, FCN2, to that of a commercial ELISA kit (Figure 5). The Spearman correlation of 0.87 demonstrates strong concordance of these two assays, particularly in the MM samples. We also confirmed the differential expression of three additional MM markers discovered in this study, CXCL13, C9 and F9 in the 62 controls and 38 MM of the validation set, with antibody-based commercial ELISA assays (Figure 6).

## Discussion

Using the SOMAscan proteomic assay, a highly sensitive candidate 13-biomarker panel was discovered and validated for the detection of MM in the asbestos-exposed population with an accuracy of 92% and detection of 88% of Stage I and II disease. The series of clinical studies encompass classifier training, verification, and validation in clinically relevant populations for the detection of MM in those at highest risk for this aggressive disease. We deliberately avoided looking for biomarkers of MM compared healthy normal controls. Many biomarker studies are initially designed to contrast the extremes of disease with healthy normals, and then when applied to the true clinical intended use the biomarkers fail. Particularly in a low prevalence disease associated with specific risk factors, identifying disease biomarkers in comparison to healthy controls is not clinically relevant.

In the next 25 years it is estimated that the diagnosis of MM will increase 5–10% each year until 2020 in most industrialized countries at a cost of $200 billion in the US and nearly $300 billion worldwide [1], [2]. The interval between asbestos exposure and the development of MM ranges from 25–71 years, yet this disease is often fatal within one year of diagnosis [1]. The large gap between asbestos exposure and disease lends itself to surveillance in the high-risk population with the goal of detecting early, treatable disease.

Since 1973 the USA Occupational Safety and Health Administration has mandated monitoring of individuals with occupational airborne asbestos exposure [2]. Monitoring currently includes chest X-ray, health history, and spirometry, but these tools are poor predictors of disease. Low dose computed tomography (LDCT) screening studies for malignant pleural mesothelioma and lung cancer in asbestos-exposed individuals have been conducted by several investigators [3], [4]. Similar to CT screening for high-risk smokers, CT scans resulted in many more cases of benign disease requiring follow-up than true malignancies detected. For example, Roberts [3] reported screening 516 asbestos-exposed individuals, resulting in a screen-detected rate of malignancy of 2.1%, while the rate for benign conditions was 70%, primarily pulmonary plaques and nodules. Only 0.6% of the study population presented with PE. Over 25% of the invasive interventions were for benign disease. There was limited benefit for detection of MM in this study, as the longest reported survival was nine months after diagnosis.

Using the same prevalence assumptions, compared to the calculated PPV/NPV of 2.5%/98.9% for initial CT screening in Robert's study and 7.2%/99.4% for mesothelin (based on performance reported in Pass et al [5]), our estimated PPV/NPV is 12.6%/99.9%. Furthermore, the accuracy of the classifier in this population is estimated at 90.8% compared to 75.9% for the initial CT screen and 88.6% for mesothelin. The classifier results would identify more true cases of MM while sending fewer individuals without MM for unnecessary followup procedures and avoiding repeated radiation exposure.

Our pre-specified classifier decision threshold ascribed equal importance to sensitivity and specificity and remained fixed throughout verification and validation. However, because MM is a rare disease even in the asbestos-exposed population, an argument could be made to assess the risk of MM at a different operating point on the ROC curve that favors specificity over sensitivity. For example, an alternative decision threshold in the validation study yielding 98% specificity in this cohort would still detect 60% of MM cases.

A potential limitation of our findings is the lack of discrimination of the classifier in patients with PE. Even if this preliminary result is confirmed in future studies, the apparent false positive rate is tolerable because the incidence of benign PE is low and detection of non-MM malignant PE is important. Roberts reported that 0.6% of their asbestos exposed screening cohort had PE while 1.4% had MM [3]. Individuals with benign PE were not excluded in our studies; therefore the background prevalence of PE in the control population is represented in the high specificity results. This non-invasive blood test could be applied as a screen for the asbestos-exposed population. Negative individuals could be spared further testing and invasive procedures while positives would be followed up with imaging to refine their diagnosis. Since most MM cases present with PE, application of this test for differential diagnosis of symptomatic individuals with PE is limited without further refinement of the classifier to improve specificity in this population.

A potential limitation of this study was the bridging between versions of the assay, which required a retraining of the classifier on the same sample set, with the exception of seven depleted samples. The new assay version resulted in a shift in the reference ranges for the biomarkers; however, the predictions produced by classifiers trained on different versions were extremely well correlated, which suggests the differential expression signatures between MM and asbestos exposed individuals were consistent between the two versions. The classifier performed well on the blinded validation set, demonstrating that the assay transition did not affect our ability to differentiate MM from controls.

To our knowledge none of the classifier biomarkers discovered in this study have been associated with MM. The list of proteins in Table 5 fall into two broad categories: inflammation and regulation of cellular proliferation. Chronic pulmonary inflammation has long been a hallmark of asbestos deposition and is thought to contribute to asbestos-related carcinogenesis. Measures of inflammation such as high neutrophil/lymphocyte ratio correlate with angiogenesis, cellular proliferation and prognosis in MM patients [21]. Consistent with these observations, over 25% of our MM candidate proteins are associated with neutrophils, leukocytes, or platelets.

Asbestos induces necrotic cell death and the accompanying release of HMGB1, which leads to chronic inflammation and accumulation of macrophage and other inflammatory cells [1], [22]. These cells release TNF-α, which activates the NF-κB pathway and increases survival of mesothelial cells after asbestos exposure, including those with asbestos-induced genetic damage that will eventually develop into malignant disease. One of our classifier proteins, TNFRSF8, is a member of the TNF receptor superfamily that mediates signal transduction leading to NF-κB activation. Eight other proteins in the classifier cluster with NF-κB in pathways involved in response to wounding and inflammation: CCL23, C9, CDK5-CDK5R1, CXCL13, F9, FCN2, FN1 and MDK [17]. Four proteins are involved in the extracellular matrix or processes regulating cell migration: CDK5-CDKR1, FN1, ICAM2, KIT, and MDK. The remaining markers function in cellular metabolism: ApoA1 and SERPINA4. Interestingly, we measure lower SERPINA4 levels in MM patients than controls, and substrates for this protease inhibitor have been reported at elevated levels in MM tissue [23]. SERPINA4, also known as kallistatin, inhibits tissue kallikrein, which promotes angiogenesis and tumor growth [24]. Thus, lower SERPINA4 may increase the availability of active tissue kallikrein and lead to angiogenesis and malignant cell proliferation [24].

Two previously described markers of MM, mesothelin and CEA, are included in our proteomic discovery array content (Table S1). Mesothelin was identified as a potential biomarker in the list of 64 candidates, (Table S1) but other markers proved to be superior by univariate analysis. In paired samples, the random forest classifier AUC of 0.99 and 91%/94% sensitivity/specificity was superior to that of mesothelin with an AUC of 0.82 and 66%/88% sensitivity/specificity, demonstrating the potential of the candidate biomarkers described here to improve detection of MM and improve patient outcomes. Differential expression of CEA was not statistically significant.

Several biologic functions are represented in the classifier, including inflammation/immune response, cell growth regulators and cellular adhesion/morphogenesis proteins. Taken together, the functions of the markers in the classifier illustrate tumor growth strategies to deregulate cellular energetics, sustain proliferation, resist cell death, and activate invasion. The supportive role of the tumor micro-environment is represented by proteins involved in avoiding immune destruction and inducing tumor-promoting inflammation. The biomarker levels correlate with pathologic stage and are a measure of disease burden as tumors evolve from local to invasive malignancy. Future studies will assess whether this correlation with tumor burden can be extended for prognosis and measuring response to therapy.

Our data suggest that the candidate markers and classifier described in this series of discovery, verification, and validation studies have the potential to improve MM surveillance and early detection, leading to more effective treatment and the potential for prolonged survival. The high specificity reduces unnecessary treatment for this rare disease, thus saving cost and reducing patient anxiety. Based on the discoveries reported here, we have initiated further validation studies in high-risk individuals for both screening and diagnosis.

## Supporting Information

Table S1 **SOMAscan protein targets and MM biomarker candidates.**
(PDF)

Table S2 **The training and verification datasets from V1 of the SOMAscan assay for the thirteen biomarkers in the panel.** The units are Relative Fluorescence Units (RFU). The Response column distinguishes the asbestos exposed samples (Control) from the malignant Mesothelioma samples (Disease). The Dataset column distinguishes between the training dataset and the verification dataset.
(XLSX)

Figure S1 **Plot of classifier prediction scores for the V1 and V2 classifier.** The plot shows consistent predictions for both models on the same 113 samples present in both versions. MM samples are colored red and asbestos exposed patients are colored green.
(DOCX)
