# Supplementary material for: Early Detection of Malignant Pleural Mesothelioma in Asbestos-Exposed Individuals with a Noninvasive Proteomics-Based Surveillance Tool
Source: PLoS One. 2012 Oct 3;7(10):e46091. doi: 10.1371/journal.pone.0046091 (PMC3463527; doi:10.1371/journal.pone.0046091)
Supplement: Table S1 — SOMAscan protein targets and MM biomarker candidates. (PDF) [file pone.0046091.s001.pdf]

Table S1: SOMAscan Protein Targets and MM Biomarker Candidates

Gene Name	GeneId	Protein Target	SwissProt ID	MM Biomarker Candidate
A2M	2	a2-Macroglobulin	P01023	
ABL1	25	ABL1	P00519	X
ABL2	27	ABL2	P42684	
ACAN	176	Aggrecan	P16112	
ACE2	59272	ACE2	Q9BYF1	
ACP1	52	PPAC	P24666	
ACP5	54	TrATPase	P13686	
ACVR1B	91	Activin RIB	P36896	
ACVRL1	94	ALK-1	P37023	
ACY1	95	Aminoacylase-1	Q03154	
ADAM12	8038	ADAM12	O43184	
ADAM9	8754	ADAM 9	Q13443	
ADAMTS1	9510	ATS1	Q9UHI8	
ADAMTS13	11093	ATS13	Q76LX8	
ADAMTS15	170689	ATS15	Q8TE58	
ADAMTS4	9507	ADAMTS-4	O75173	
ADAMTS5	11096	ADAMTS-5	Q9UNA0	
ADCYAP1	116	PACAP-27	P18509	
ADCYAP1	116	PACAP-38	P18509	
ADIPOQ	9370	Adiponectin	Q15848	
ADRBK1	156	BARK1	P25098	
ADSL	158	PUR8	P30566	
AFM	173	Afamin	P43652	X
AGER	177	sRAGE	Q15109	
AGR2	10551	AGR2	O95994	
AGRP	181	ART	O00253	
AGT	183	Angiotensinogen	P01019	
AHCY	191	SAHH	P23526	
AHSG	197	a2-HS-Glycoprotein	P02765	
AIF1	199	AIF1	P55008	
AIMP1	9255	EMAP-2	Q12904	
AIP	9049	AIP	O00170	
AK1	203	Myokinase, human	P00568	
AKR1A1	10327	AK1A1	P14550	
AKR7A2	8574	Aflatoxin B1 aldehyde reductase	O43488	
AKT1	207	PKB	P31749	
AKT3	10000	PKB gamma	Q9Y243	
ALB	213	Albumin	P02768	X
ALCAM	214	ALCAM	Q13740	
ALDOA	226	aldolase A	P04075	
ALPL	249	Alkaline phosphatase, bone	P05186	X
AMH	268	MIS	P03971	

Gene Name	GeneId	Protein Target	SwissProt ID	MM Biomarker Candidate
AMN	81693	AMNLS	Q9BXJ7	
ANG	283	Angiogenin	P03950	
ANGPT1	284	Angiopoietin-1	Q15389	
ANGPT2	285	Angiopoietin-2	O15123	
ANGPTL3	27329	ANGL3	Q9Y5C1	
ANGPTL4	51129	ANGL4	Q9BY76	
ANXA1	301	annexin I	P04083	
ANXA2	302	annexin II	P07355	
APCS	325	SAP	P02743	
APOA1	335	Apo A-I	P02647	X
APOB	338	Apo B	P04114	
APOD	347	Apo D	P05090	
APOE	348	Apo E	P02649	
APOE	348	Apo E3	P02649	
APOE	348	Apo E4	P02649	
APP	351	amyloid precursor protein	P05067	
AREG	374	AREG	P15514	
ARG1	383	ARGI1	P05089	
ARID3A	1820	ARI3A	Q99856	
ARPP19	10776	ARP19	P56211	
ARSA	410	Arylsulfatase A	P15289	
ARSB	411	ARSB	P15848	
ARTN	9048	Artemin	Q5T4W7\r\n	
ASAH2	56624	ASAH2	Q9NR71	
ASGR1	432	ASGR1	P07306	
ATP5B	506	ATP synthase beta chain	P06576	
AURKA	6790	Aurora kinase A	O14965	
AURKB	9212	AURKB	Q96GD4	
AZU1	566	Azurocidin	P20160	X
B2M	567	b2-Microglobulin	P61769	
BAD	572	BAD	Q92934	
BCAM	4059	BCAM	P50895	
BCAN	63827	PGCB	Q96GW7	
BCL2	596	Bcl-2	P10415	
BCL2A1	597	BFL1	Q16548	
BCL2L1	598	BCL2-like 1 protein	Q07817	
BDNF	627	BDNF	P23560	X
BGN	633	BGN	P21810	
BID	637	BID	P55957	
BIRC3	330	cIAP-2	Q13489	
BIRC5	332	Survivin	O15392	
BIRC7	79444	Livin B	Q96CA5	
BMP1	649	BMP-1	P13497	X
BMP10	27302	BMP10	O95393	

Gene Name	GeneId	Protein Target	SwissProt ID	MM Biomarker Candidate
BMP6	654	BMP-6	P22004	
BMP7	655	BMP-7	P18075	
BMPER	168667	BMPER	Q8N8U9	X
BMPR1A	657	BMPR1A	P36894	
BMPR2	659	BMP RII	Q13873	
BMX	660	BMX	P51813	X
BOC	91653	BOC	Q9BWV1	
BPI	671	BPI	P17213	X
BSG	682	BASI	P35613	
BST1	683	BST1	Q10588	
BTK	695	BTK	Q06187	
C14orf166	51637	CN166	Q9Y224	
C1QA C1QB C1QC	712 713 714	C1q	P02745, P02746, P02747	
C1QBP	708	C1QBP	Q07021	
C1R	715	C1r	P00736	
C1S	716	C1s	P09871	
C2	717	C2	P06681	
C3	718	C3	P01024	
C3	718	C3a	P01024	
C3	718	C3adesArg	P01024	
C3	718	C3b	P01024	
C3	718	C3d	P01024	
C3	718	C3d	P01024	
C3	718	iC3b	P01024	
C4A C4B	720 721	C4	P0C0L4, P0C0L5	
C4A C4B	720 721	C4b	P0C0L4, P0C0L5	
C5	727	C5	P01031	
C5	727	C5a	P01031	
C5 C6	727 729	C5b, 6 Complex	P01031, P13671	
C6	729	C6	P13671	
C7	730	C7	P10643	
C8A C8B C8G	731 732 733	C8	P07357, P07358, P07360	
C9	735	C9	P02748	X
CA1	759	Carbonic anhydrase I	P00915	
CA10	56934	Carbonic Anhydrase X	Q9NS85	
CA13	377677	Carbonic anhydrase XIII	Q8N1Q1	
CA2	760	carbonic anhydrase II	P00918	
CA3	761	Carbonic anhydrase III	P07451	
CA4	762	Carbonic Anhydrase IV	P22748	
CA6	765	Carbonic anhydrase 6	P23280	
CA7	766	Carbonic anhydrase VII	P43166	
CA9	768	Carbonic anhydrase 9	Q16790	

Gene Name	GeneId	Protein Target	SwissProt ID	MM Biomarker Candidate
CADM1	23705	Nectin-like protein 2	Q9BY67	
CADM3	57863	Nectin-like protein 1	Q8N126	
CAMK1	8536	CAMK1	Q14012	X
CAMK1D	57118	CAMK1D	Q8IU85	
CAMK2A	815	CAMK2A	Q9UQM7	
CAMK2B	816	CAMK2B	Q13554	
CAMK2D	817	CAMK2D	Q13557	
CAMKK1	84254	CaMKK alpha	Q8N5S9	
CAPG	822	CAPG	P40121	
CAPN1 CAPNS1	823 826	Calpain I	P07384, P04632	
CASP2	835	Caspase-2	P42575	
CASP3	836	Caspase-3	P42574	
CAST	831	Calpastatin	P20810	
CAT	847	Catalase	P04040	
CBX5	23468	CBX5	P45973	
CCDC80	151887	URB	Q76M96	X
CCL1	6346	I-309	P22362	
CCL13	6357	MCP-4	Q99616	
CCL14	6358	HCC-1	Q16627	
CCL15	6359	MIP-5	Q16663	
CCL16	6360	HCC-4	O15467	
CCL17	6361	TARC	Q92583	
CCL18	6362	PARC	P55774	
CCL19	6363	MIP-3b	Q99731	
CCL2	6347	MCP-1	P13500	
CCL20	6364	MIP-3a	P78556	
CCL21	6366	6Ckine	O00585	
CCL22	6367	MDC	O00626	
CCL23	6368	Ck-b-8-1	P55773	X
CCL23	6368	MPIF-1	P55773	X
CCL24	6369	Eotaxin-2	O00175	
CCL25	6370	TECK	O15444	
CCL27	10850	CTACK	Q9Y4X3	
CCL28	56477	CCL28	Q9NRJ3	
CCL3	6348	MIP-1a	P10147	
CCL3L1	414062	LD78-beta	P16619	
CCL4	388372	MIP-1b	P13236	
CCL4L1	388372	LAG-1	Q8NHW4	
CCL5	6352	RANTES	P13501	
CCL7	6354	MCP-3	P80098	
CCL8	6355	MCP-2	P80075	
CD109	135228	CD109	Q6YHK3	
CD163	9332	sCD163	Q86VB7	
CD207	50489	CLC4K	Q9UJ71	
CD209	30835	DC-SIGN	Q9NNX6	

Gene Name	GeneId	Protein Target	SwissProt ID	MM Biomarker Candidate
CD22	933	CD22	P20273	
CD33	945	Siglec-3	P20138	
CD36	948	CD36 ANTIGEN	P16671	
CD4	920	sCD4	P01730	
CD40LG	959	CD40 ligand, soluble	P29965	
CD48	962	CD48	P09326	
CD5L	922	CD5L	O43866	
CD70	970	CD70	P32970	
CD80	941	B7	P33681	
CD84	8832	SLAF5	Q9UIB8	
CD97	976	CD97	P48960	
CDC2 CCNB1	983 891	CDK1/cyclin B	P06493, P14635	
CDC37	11140	CDC37	Q16543	
CDC42BPB	9578	MRCKB	Q9Y5S2	
CDH1	999	Cadherin E	P12830	X
CDH12	1010	Cadherin-12	P55289	
CDH2	1000	Cadherin-2	P19022	
CDH3	1001	P-Cadherin	P22223	
CDH5	1003	Cadherin-5	P33151	
CDH6	1004	Cadherin-6	P55285	
CDK2 CCNA2	1017 890	CDK2/cyclin A	P24941, P20248	
CDK5 CDK5R1	1020 1775	CDK5/p35	Q00535, Q15078	X
CDK8 CCNC	1024 892	CDK8/cyclin C	P49336, P24863	X
CDKN1B	1027	p27Kip1	P46527	
CDNF	441549	ARMEL	Q49AH0	
CDON	50937	CDON	Q4KMG0	
CEACAM5	1048	CEA	P06731	
CFB	629	Factor B	P00751	
CFC1	55997	CFC1	Q9GZR3	
CFD	1675	Factor D	P00746	
CFH	3075	Factor H	P08603	
CFHR5	81494	complement factor H-related 5	Q9BXR6	X
CFI	3426	Factor I	P05156	
CFL1	1072	Cofilin-1	P23528	X
CFP	5199	Properdin	P27918	X
CGA CGB	1081 1082	HCG	P01215, P01233	
CGA FSHB	1081 2488	FSH	P01215, P01225	
CGA LHB	1081 3972	Luteinizing hormone	P01215, P01229	
CGA TSHB	1081 12372	TSH	P01215, P01222	
CHEK1	1111	CHK1	O14757	
CHEK2	11200	Chk2	O96017	
CHI3L1	1116	YKL-40	P36222	
CHIT1	1118	Chitotriosidase-1	Q13231	
CHL1	10752	CHL1	O00533	
CHRDL1	91851	CRDL1	Q9BU40	

Gene Name	GeneId	Protein Target	SwissProt ID	MM Biomarker Candidate
CHST15	51363	ST4S6	Q7LFX5	
CHST2	9435	CHST2	Q9Y4C5	
CHST6	4166	CHST6	Q9GZX3	
CKB	1152	CK-BB	P12277	
CKB CKM	1152 1158	CK-MB	P12277, P06732	
CKM	1158	CK-MM	P06732	
CLEC11A	6320	SCGF-alpha	Q9Y240	
CLEC11A	6320	SCGF-beta	Q9Y240	
CLEC1B	51266	CLC1B	Q9P126	
CLEC4M	10332	DC-SIGNR	Q9H2X3	
CLEC7A	64581	CLC7A	Q9BXN2	
CLIC1	1192	NCC27	O00299	
CLU	1191	Clusterin	P10909	
CMA1	1215	Chymase	P23946	
CMPK1	51727	Cytidylate kinase	P30085	
CNDP1	84735	CNDP1	Q96KN2	
CNDP2	55748	Glutamate carboxypeptidase	Q96KP4	
CNTF	1270	CNTF	P26441	
CNTFR	1271	CNTFR alpha	P26992	
CNTN1	1272	contactin-1	Q12860	
CNTN2	6900	CNTN2	Q02246	
CNTN4	152330	Contactin-4	Q8IWV2	
CNTN5	53942	Contactin-5	O94779	
COL18A1	80781	Endostatin	P39060	
COL23A1	91522	CONA1	Q86Y22	
COL8A1	1295	CO8A1	P27658	
COLEC11	78989	Collectin Kidney 1	Q9BWP8	
COLEC12	81035	COLEC12	Q5KU26	
COMMD7	149951	COMMD7	Q86VX2	
COTL1	23406	Coactosin-like protein	Q14019	
CPB2	1361	TAFI	Q96IY4	
CRISP3	10321	CRIS3	P54108	
CRK	1398	CRK	P46108	X
CRLF1 CLCF1	9244 23529	CLF-1/CLC Complex	O75462, Q9UBD9	
CRLF2	64109	TSLP R	Q9HC73	
CRP	1401	CRP	P02741	X
CSF1	1435	CSF-1	P09603	
CSF1R	1436	M-CSF R	P07333	
CSF2	1437	GM-CSF	P04141	
CSF3	1440	G-CSF	P09919	
CSF3R	1441	G-CSF-R	Q99062	
CSK	1445	CSK	P41240	
CSN1S1	1446	CASA	P47710	X
CSNK2A1	1457	CSK21	P68400	
CST1	1469	CYTN	P01037	

Gene Name	GeneId	Protein Target	SwissProt ID	MM Biomarker Candidate
CST2	1470	CYTT	P09228	
CST3	1471	Cystatin C	P01034	
CST5	1473	CYTD	P28325	
CST6	1474	Cystatin M	Q15828	
CST7	8530	CYTF	O76096	
CTF1	1489	Cardiotrophin-1	Q16619	
CTGF	1490	CTGF	P29279	
CTLA4	1493	CTLA-4	P16410	
CTSA	5476	Cathepsin A	P10619	
CTSB	1508	Cathepsin B	P07858	
CTSC	1075	CATC	P53634	
CTSD	1509	Cathepsin D	P07339	
CTSE	1510	CATE	P14091	
CTSG	1511	Cathepsin G	P08311	
CTSH	1512	Cathepsin H	P09668	
CTSL2	1515	Cathepsin V	O60911	
CTSS	1520	Cathepsin S	P25774	
CTSZ	1522	CATZ	Q9UBR2	
CX3CL1	6376	Fractalkine/CX3CL-1	P78423	
CXCL1	2919	Gro-a	P09341	
CXCL10	3627	IP-10	P02778	
CXCL11	6373	I-TAC	O14625	
CXCL12	6387	SDF-1a	P48061	
CXCL12	6387	SDF-1b	P48061	
CXCL13	10563	BLC	O43927	X
CXCL16	58191	CXCL16, soluble	Q9H2A7	
CXCL2	2920	Gro-b	P19875	
CXCL3	2921	Gro-g	P19876	
CXCL5	6374	ENA-78	P42830	
CXCL6	6372	GCP-2	P80162	
CYCS	54205	Cytochrome c	P99999	
CYP3A4	1576	Cytochrome P450 3A4	P08684	
DAPK2	23604	DAPK2	Q9UIK4	
DCN	1634	Bone proteoglycan II	P07585	
DCTN2	10540	Dynactin subunit 2	Q13561	
DCTPP1	79077	XTP3A	Q9H773	
DDC	1644	dopa decarboxylase	P20711	X
DDR1	780	discoidin domain receptor 1	Q08345	
DDR2	4921	Discoidin domain receptor 2	Q16832	
DDX19B	11269	DEAD-box protein 19B	Q9UMR2	
DHH	50846	DHH	O43323	
DHH	50846	DHH	O43323	
DIABLO	56616	SMAC	Q9NR28	
DKK1	22943	DKK1	O94907	
DKK3	27122	DKK3	Q9UBP4	

Gene Name	GeneId	Protein Target	SwissProt ID	MM Biomarker Candidate
DKK4	27121	Dkk-4	Q9UBT3	
DKKL1	27120	Soggy-1	Q9UK85	
DLL4	54567	DLL4	Q9NR61	
DMP1	1758	DMP1	Q13316	
DNAJB1	3337	HSP 40	P25685	
DNAJC19	131118	DnaJ homolog	Q96DA6	
DPP7	29952	DPP2	Q9UHL4	
DSC3	1825	DSC3	Q14574	
DSG1	1828	Desmoglein-1	Q02413	
DUSP3	1845	DUS3	P51452	
DYNLL1	8655	DLC8	P63167	
DYNLRB1	83658	DLRB1	Q9NP97	
DYRK3	8444	DYRK3	O43781	
ECE1	1889	Endothelin-converting enzyme 1	P42892	
ECM1	1893	ECM1	Q16610	
EDA	1896	EDA	Q92838	
EDA2R	60401	XEDAR	Q9HAV5	
EDAR	10913	EDAR	Q9UNE0	
EEF1B2	1933	EF-1-beta	P24534	
EFNA4	1945	Ephrin-A4	P52798	
EFNA5	1946	Ephrin-A5	P52803	X
EFNB3	1949	Ephrin-B3	Q15768	
EGFR	1956	ERBB1	P00533	X
EHMT2	10919	NG36	Q96KQ7	
EIF4A3	9775	IF4A3	P38919	
EIF4EBP2	1979	4EBP2	Q13542	X
EIF4G2	1982	IF4G2	P78344	
EIF4H	7458	eIF-4H	Q15056	
EIF5	1983	eIF-5	P55010	
EIF5A	1984	eIF-5A-1	P63241	
EMR2	30817	EMR2	Q9UHX3	
ENG	2022	Endoglin	P17813	
ENPP7	339221	ENPP7	Q6UWV6	
ENTPD1	953	CD39	P49961	
ENTPD3	956	ENTP3	O75355	
ENTPD5	957	ENTP5	O75356	
EPB41	2035	41	P11171	
EPHA1	2041	EphA1	P21709	
EPHA2	1969	Epithelial cell kinase	P29317	
EPHA3	2042	EPHA3	P29320	
EPHA5	2044	EphA5	P54756	
EPHB4	2050	EphB4	P54760	
EPO	2056	Epo	P01588	
EPOR	2057	EPO-R	P19235	
EPS15L1	58513	EP15R	Q9UBC2	

Gene Name	GeneId	Protein Target	SwissProt ID	MM Biomarker Candidate
ERAP1	51752	ARTS1	Q9NZ08	
ERBB2	2064	ERBB2	P04626	
ERBB3	2065	ERBB3	P21860	
ERBB4	2066	ERBB4	Q15303	
EREG	2069	EPI	O14944	
ERP29	10961	ERP29	P30040	
ESAM	90952	ESAM	Q96AP7	
ESD	2098	Esterase D	P10768	
ESM1	11082	Endocan	Q9NQ30	X
ESR1	2099	ER	P03372	
ETHE1	23474	ETHE1	O95571	
F10	2159	Coagulation Factor X	P00742	
F10	2159	Coagulation Factor X	P00742	
F10	2159	Coagulation Factor Xa	P00742	
F11	2160	Coagulation Factor XI	P03951	
F2	2147	Thrombin	P00734	
F3	2152	TF	P13726	
F5	2153	Coagulation Factor V	P12259	
F5	2153	Coagulation Factor V	P12259	
F7	2155	Coagulation Factor VII	P08709	
F9	2158	Coagulation Factor IX	P00740	
F9	2158	Coagulation Factor IX	P00740	X
FABP3	2170	FABP	P05413	
FABP5	2171	FABPE	Q01469	
FAM107A	11170	DRR1	O95990	
FAM107B	83641	FAM107B	Q9H098	
FAP	2191	SEPR	Q12884	
FASLG	356	Fas ligand, soluble	P48023	
FCAR	2204	FCAR	P24071	
FCER2	2208	CD23	P06734	
FCGR1A	2209	FCGR1	P12314	
FCGR2A	2212	FCG2A	P12318	
FCGR2B	2213	FCG2B	P31994	
FCGR3B	2215	FCG3B	O75015	
FCN1	2219	FCN1	O00602	
FCN2	2220	FCN2	Q15485	X
FCN3	8547	Ficolin-3	O75636	
FCRL3	115352	FCRL3	Q96P31	
FER	2241	FER	P16591	
FETUB	26998	FETUB	Q9UGM5	
FGA FGB FGG	2243 2244 2266	D-dimer	P02671, P02675, P02679	X
FGA FGB FGG	2243 2244 2266	Fibrinogen	P02671, P02675, P02679	
FGF1	2246	b-ECGF	P05230	

Gene Name	GeneId	Protein Target	SwissProt ID	MM Biomarker Candidate
FGF10	2255	FGF-10	O15520	
FGF12	2257	FGF-12	P61328	
FGF16	8823	FGF-16	O43320	
FGF17	8822	FGF-17	O60258	
FGF18	8817	FGF-18	O76093	
FGF19	9965	FGF-19	O95750	
FGF2	2247	bFGF	P09038	
FGF20	26281	FGF-20	Q9NP95	
FGF23	8074	FGF23	Q9GZV9	
FGF4	2249	FGF-4	P08620	
FGF5	2250	FGF-5	P12034	
FGF6	2251	FGF-6	P10767	
FGF7	2252	FGF7	P21781	
FGF8	2253	FGF-8A	P55075	
FGF8	2253	FGF-8B	P55075	
FGF9	2254	FGF9	P31371	
FGFR1	2260	bFGF-R	P11362	
FGFR2	2263	FGFR-2	P21802	
FGFR3	2261	FGFR-3	P22607	
FGG	2266	Fibrinogen g-chain dimer	P02679	
FGR	2268	FGR	P09769	
FIGF	2277	VEGF-D	O43915	
FLRT1	23769	FLRT1	Q9NZU1	
FLT3	2322	Flt-3	P36888	
FLT3LG	2323	Flt3 ligand	P49771	X
FLT4	2324	VEGF sR3	P35916	
FN1	2335	Fibronectin	P02751	X
FN1	2335	FN1.3	P02751	
FN1	2335	FN1.4	P02751	X
FOLH1	2346	PSMA	Q04609	
FRZB	2487	sFRP-3	Q92765	X
FSCN1	6624	Fascin	Q16658	
FST	10468	FST	P19883	
FSTL3	10272	FSTL3	O95633	
FTH1 FTL	2495 2512	Ferritin	P02794, P02792	
FUT3	2525	Fucosyltransferase 3	P21217	
FUT5	2527	FUT5	Q11128	
FYN	2534	FYN	P06241	
FYN	2534	FYN	P06241	
GAPDH	2597	GAPDH, liver	P04406	
GAS1	2619	GAS1	P54826	
GCG	2641	Glucagon	P01275	
GDF11	10220	GDF-11	O95390	
GDF2	2658	GDF2	Q9UK05	
GDF5	8200	BMP-14	P43026	

Gene Name	GeneId	Protein Target	SwissProt ID	MM Biomarker Candidate
GDF9	2661	GDF-9	O60383	
GDI2	2665	Rab GDP dissoc inhibitor beta	P50395	
GDNF	2668	GDNF	P39905	
GFAP	2670	GFAP	P14136	
GFRA1	2674	GFRa-1	P56159	
GFRA2	2675	GFRa-2	O00451	
GFRA3	2676	GFRa-3	O60609	
GHR	2690	Growth hormone receptor	P10912	
GNB2L1	10399	RACK1	P63244	
GNLY	10578	Granulysin	P22749	
GNS	2799	GNS	P15586	
GOT1	2805	GOT1	P17174	
GP1BA	2811	GP1BA	P07359	
GP6	51206	GPVI	Q9HCN6	
GPC2	221914	GPC2	Q8N158	X
GPC3	2719	Glypican 3	P51654	
GPC5	2262	GPC5	P78333	
GPI	2821	PHI	P06744	X
GPR114	221188	GP114	Q8IZF4	
GPRASP2	114928	GASP-2	Q96D09	
GPT	2875	ALT	P24298	
GRAP2	9402	GRB2-related adapter protein 2	O75791	
GREM1	26585	GREM1	O60565	
GRN	2896	GRN	P28799	
GRP	2922	Gastrin-releasing peptide	P07492	
GSK3A	2931	GSK-3 alpha	P49840	
GSK3B	2932	GSK-3 beta	P49841	
GSN	2934	Gelsolin	P06396	
GSTA3	2940	GSTA3	Q16772	
GZMA	3001	granzyme A	P12544	
GZMB	3002	Granzyme B	P10144	
GZMH	2999	Granzyme H	P20718	
H2AFZ	3015	Histone H2A.z	P0C0S5	X
HAAO	23498	3HAO	P46952	
HAMP	57817	LEAP-1	P81172	
HAPLN1	1404	HPLN1	P10915	
HAT1	8520	Hat1	O14929	
HBA1 HBB	3039 3043	Hemoglobin	P69905, P68871	
HBEGF	1839	HB-EGF	Q99075	
HCK	3055	HCK	P08631	
HDAC8	55869	HDAC8	Q9BY41	
HDGFRP2	84717	HDGR2	Q7Z4V5	
HFE2	148738	RGM-C	Q6ZVN8	
HGF	3082	HGF	P14210	
HGFAC	3083	HGFA	Q04756	

Gene Name	GeneId	Protein Target	SwissProt ID	MM Biomarker Candidate
HIBADH	11112	3HIDH	P31937	
HINT1	3094	HINT1	P49773	X
HIPK3	10114	HIPK3	Q9H422	
HIST1H1C	3006	Histone H1.2	P16403	
HLA-DRB1	3123	2B11	P04229, P13760	
HMGB1	3146	HMG-1	P09429	
HMOX2	3163	HO-2	P30519	
HNRNPAB	3182	hnRNP A/B	Q99729	
HNRNPK	3190	hnRNP K	P61978	
HP	3240	Haptoglobin, Mixed Type	P00738	
HPGD	3248	HPG-	P15428	
HPX	3263	Hemopexin	P02790	
HRG	3273	HRG	P04196	
HS6ST1	9394	H6ST1	O60243	
HSD17B1	3292	17-beta-HSD 1	P14061	
HSD17B10	3028	ERAB	Q99714	
HSP90AA1	3320	HSP 90a	P07900	
HSP90AB1	3326	HSP 90b	P08238	
HSPA1A	3303	HSP 70	P08107	
HSPA8	3312	HSP70 protein 8	P11142	
HSPB1	3315	HSP 27	P04792	
HSPD1	3329	HSP 60	P10809	
HTRA2	27429	HTRA2	O43464	
Human-virus	1489079	HPV E7 Type 16	P03129	
Human-virus	1489089	HPV E7 Type18	P06788	
Human-virus	1724716	HIV-2 Rev	P18093	
Human-virus	None	C34 gp41 HIV Fragment	Q70626	
IBSP	3381	BSP	P21815	
ICAM1	3383	sICAM-1	P05362	
ICAM2	3384	sICAM-2	P13598	X
ICAM3	3385	sICAM-3	P32942	
ICOS	29851	ICOS	Q9Y6W8	
IDE	3416	IDE	P14735	
IDS	3423	IDS	P22304	
IDUA	3425	IDUA	P35475	
IFNA2	3440	IFN-aA	P01563	
IFNG	3458	IFN-g	P01579	
IFNGR1	3459	IFN-g R1	P15260	
IGF1	3479	IGF-I	P05019	
IGF1R	3480	IGF-I sR	P08069	
IGF2R	3482	IGF-II receptor	P11717	
IGFBP1	3484	IGFBP-1	P08833	
IGFBP2	3485	IGFBP-2	P18065	
IGFBP3	3486	IGFBP-3	P17936	
IGFBP4	3487	IGFBP-4	P22692	

Gene Name	GeneId	Protein Target	SwissProt ID	MM Biomarker Candidate
IGFBP5	3488	IGFBP-5	P24593	
IGFBP6	3489	IGFBP-6	P24592	
IGFBP7	3490	IGFBP-7	Q16270	
IGHD IGK@ IGL@	3495 50802 3535	IgD	P01880	
IGHE IGK@ IGL@	3497 50802 3535	IgE	P01854	
IGHG1 IGHG2 IGHG3 IGHG4 IGK@ IGL@	3500 3501 3502 3503 50802 3535	IgG	P01857	
IGHG1 IGHG2 IGHG3 IGHG4 IGK@ IGL@	3500 3501 3502 3503 50802 3535	IgG	P01857	
IGHM IGJ IGK@ IGL@	3507 3512 50802 3535	IgM	P01871	
IL10	3586	IL-10	P22301	
IL10RB	3588	IL-10 Rb	Q08334	
IL11	3589	IL-11	P20809	
IL11RA	3590	IL-11 RA	Q14626	
IL12A IL12B	3592 3593	IL-12	P29459, P29460	
IL12B IL23A	3593 51561	IL-23	P29460, Q9NPF7	
IL12RB1	3594	IL-12 Rb1	P42701	
IL12RB2	3595	IL-12 RB2	Q99665	
IL13	3596	IL-13	P35225	
IL13RA1	3597	IL-13 Ra1	P78552	
IL15RA	3601	IL-15 Ra	Q13261	
IL16	3603	IL-16	Q14005	
IL17A	3605	IL-17	Q16552	
IL17B	27190	IL-17B	Q9UHF5	
IL17D	53342	IL-17D	Q8TAD2	
IL17F	112744	IL-17F	Q96PD4	
IL17RA	23765	IL-17 sR	Q96F46	
IL17RC	84818	IL-17 RC	Q8NAC3	
IL17RD	54756	IL-17 RD	Q8NFM7	
IL18BP	10068	IL-18 BPa	O95998	
IL18R1	8809	IL-18 Ra	Q13478	
IL18RAP	8807	IL-18 Rb	O95256	
IL19	29949	IL-19	Q9UHD0	
IL1A	3552	IL-1a	P01583	
IL1B	3553	IL-1b	P01584	
IL1F7	27178	IL-1F7	Q9NZH6	
IL1R1	3554	IL-1 sRI	P14778	
IL1RAP	3556	IL-1 R AcP	Q9NPH3	
IL1RL1	9173	IL-1 R4	Q01638	
IL1RL2	8808	IL-1Rrp2	Q9HB29	

Gene Name	GeneId	Protein Target	SwissProt ID	MM Biomarker Candidate
IL2	3558	IL-2	P60568	
IL20	50604	IL-20	Q9NYY1	
IL22	50616	IL-22	Q9GZX6	
IL22RA1	58985	IL22RA1	Q8N6P7	
IL24	11009	IL24	Q13007	
IL25	64806	IL-17E	Q9H293	
IL27	246778	IL-27	Q8NEV9	
IL28A	282616	IFN-lambda 2	Q8IZJ0	
IL29	282618	IFN-lambda 1	Q8IU54	
IL2RA	3559	IL-2 sRa	P01589	
IL2RG	3561	IL-2 sRg	P31785	
IL3	3562	IL-3	P08700	
IL31	386653	IL-31	Q6EBC2	X
IL34	146433	IL-34	Q6ZMJ4	
IL3RA	3563	IL-3 Ra	P26951	
IL4	3565	IL-4	P05112	
IL4R	3566	IL-4 sR	P24394	
IL5	3567	IL-5	P05113	
IL5RA	3568	IL-5 Ra	Q01344	
IL6	3569	IL-6	P05231	
IL6R	3570	IL-6 sRa	P08887	
IL6ST	3572	gp130, soluble	P40189	
IL7	3574	IL-7	P13232	
IL7R	3575	IL-7 Ra	P16871	
IL8	3576	IL-8	P10145	
ING1	3621	ING1	Q9UK53	
INHBA	3624	Activin A	P08476	
INHBA INHBB	3624 3625	Activin AB	P08476, P09529	
INSR	3643	IR	P06213	
ITGA1 ITGB1	3672 3688	Integrin a1b1	P56199, P05556	X
ITGA2B ITGB3	3674 3690	gpIIbIIIa	P08514, P05106	
ITGAV ITGB5	3685 3693	Integrin aVb5	P06756, P18084	
ITIH4	3700	ITI heavy chain H4	Q14624	X
JAK2	3717	JAK2	O60674	
JAM2	58494	JAM-B	P57087	
JAM3	83700	JAM-C	Q9BX67	
KDR	3791	VEGF sR2	P35968	
KIRREL3	84623	KIRR3	Q8IZU9	
KIT	3815	SCF sR	P10721	X
KLK11	11012	Kallikrein 11	Q9UBX7	
KLK12	43849	kallikrein 12	Q9UKR0	
KLK14	43847	kallikrein 14	Q9P0G3	
KLK3	354	PSA	P07288	
KLK3	354	PSA	P07288	
KLK3 SERPINA3	354 12	PSA-ACT	P07288, P01011	X

Gene Name	GeneId	Protein Target	SwissProt ID	MM Biomarker Candidate
KLK4	9622	Kallikrein 4	Q9Y5K2	
KLK5	25818	kallikrein 5	Q9Y337	
KLK6	5653	Kallikrein 6	Q92876	
KLK7	5650	Kallikrein 7	P49862	
KLK8	11202	kallikrein 8	O60259	
KLKB1	3818	Prekallikrein	P03952	
KLRK1	22914	NKG2D	P26718	
KNG1	3827	Kininogen, HMW, Single Chain	P01042	
KNG1	3827	Kininogen, HMW, Single Chain	P01042	
KNG1	3827	Kininogen, HMW, Two Chain	P01042	
KPNA2	3838	Karyopherin-a2	P52292	
KPNB1	3837	IMB1	Q14974	
KREMEN2	79412	KREM2	Q8NCW0	
KYNU	8942	KYNU	Q16719	
L1CAM	3897	NCAM-L1	P32004	
LAMA1 LAMB1 LAMC1	284217 3912 3915	Laminin	P25391, P07942, P11047	
LAYN	143903	Layilin	Q6UX15	
LBP	3929	LBP	P18428	
LCK	3932	LCK	P06239	
LCK	3932	LCK	P06239	
LCMT1	51451	LCMT1	Q9UIC8	
LCN2	3934	Lipocalin 2	P80188	X
LCORL	254251	transcription factor MLR1, isoform CRA_b	Q8N3X6	
LDHB	3945	LDH-H 1	P07195	
LEP	3952	Leptin	P41159	
LGALS2	3957	Galectin-2	P05162	
LGALS3	3958	Galectin-3	P17931	
LGALS3BP	3959	LG3BP	Q08380	
LGALS4	3960	Galectin-4	P56470	
LGALS8	3964	Galectin-8	O00214	
LGMN	5641	LGMN	Q99538	
LIFR	3977	LIF sR	P42702	
LIN7B	64130	LIN7B	Q9HAP6	
LMNB1	4001	Lamin-B1	P20700	
LPO	4025	PERL	P22079	
LRIG3	121227	LRIG3	Q6UXM1	
LRP8	7804	LRP8	Q14114	
LRPAP1	4043	RAP	P30533	
LRRTM1	347730	LRRT1	Q86UE6	
LRRTM3	347731	LRRT3	Q86VH5	
LSAMP	4045	LSAMP	Q13449	
LTA	4049	TNF-b	P01374	
LTA LTB	4049 4050	Lymphotoxin a1/b2	P01374, Q06643	

Gene Name	GeneId	Protein Target	SwissProt ID	MM Biomarker Candidate
LTA LTB	4049 4050	Lymphotoxin a2/b1	P01374, Q06643	
LTA4H	4048	LKHA4	P09960	
LTBR	4055	Lymphotoxin b R	P36941	
LTF	4057	Lactoferrin	P02788	X
LY86	9450	LY86	O95711	
LY9	4063	LY9	Q9HBG7	
LYN	4067	LYN	P07948	
LYN	4067	LYNB	P07948	
LYVE1	10894	LYVE1	Q9Y5Y7	
LYZ	4069	Lysozyme	P61626	
MAP2K1	5604	MEK1	Q02750	
MAP2K2	5605	MP2K2	P36507	
MAP2K4	6416	MP2K4	P45985	
MAPK1	5594	MK01	P28482	
MAPK11	5600	MK11	Q15759	
MAPK12	6300	MK12	P53778	
MAPK13	5603	MK13	O15264	
MAPK14	1432	MAPK14	Q16539	
MAPK3	5595	ERK-1	P27361	
MAPK8	5599	MK08	P45983	
MAPKAPK2	9261	MAPK2	P49137	
MAPKAPK3	7867	MAPKAPK3	Q16644	
MAPT	4137	tau	P10636	
MASP1	5648	MASP3	P48740	
MATK	4145	MATK	P42679	
MATN2	4147	MATN2	O00339	
MATN3	4148	MATN3	O15232	
MB	4151	Myoglobin	P02144	
MBD4	8930	MBD4	O95243	
MBL2	4153	MBL	P11226	
MCM2	4171	MCM2	P49736	
MDH1	4190	MDHC	P40925	
MDK	4192	Midkine	P21741	X
MDM2	4193	MDM2	Q00987	
MED1	5469	MED-1	Q15648	
MEPE	56955	MEPE	Q9NQ76	
MET	4233	Met	P08581	
METAP1	23173	METAP1	P53582	
METAP2	10988	AMPM2	P50579	
MFGE8	4240	MFGM	Q08431	
MFRP	114902	MFRP	Q9BY79	
MIA	8190	MIA	Q16674	
MICA	4276	MICA	Q29983	
MMEL1	79258	MMEL2	Q495T6	
MMP1	4312	MMP-1	P03956	

Gene Name	GeneId	Protein Target	SwissProt ID	MM Biomarker Candidate
MMP10	4319	MMP-10	P09238	
MMP12	4321	MMP-12	P39900	
MMP13	4322	MMP-13	P45452	
MMP14	4323	MMP-14	P50281	
MMP2	4313	MMP-2	P08253	
MMP3	4314	MMP-3	P08254	
MMP7	4316	MMP-7	P09237	
MMP8	4317	MMP-8	P22894	
MMP9	4318	MMP-9	P14780	X
MPL	4352	Thrombopoietin Receptor	P40238	
MPO	4353	Myeloperoxidase	P05164	X
MRC1	4360	Macrophage mannose receptor	P22897	
MRC2	9902	MRC2	Q9UBG0	
MSLN	10232	Mesothelin	Q13421	X
MSLN	10232	Mesothelin	Q13421	
MSN	4478	Moesin	P26038	
MSR1	4481	Macrophage scavenger receptor	P21757	
MST1	4485	MSP	P26927	
MST1R	4486	MSP R	Q04912	
MYL2	4633	Myosin regulatory light chain 2	P10916	
MYST3	7994	MOZ	Q92794	
NAAA	27163	ASAHL	Q02083	
NACA	4666	NACA	Q13765	
NAGK	55577	NAGK	Q9UJ70	
NAMPT	10135	PBEF	P43490	
NANOG	79923	NANOG	Q9H9S0	
NAPA	8775	SNAA	P54920	
NBL1	4681	DAN	P41271	
NCAM1	4684	NCAM-120	P13591	
NCK1	4690	NCK1	P16333	
NCR2	9436	NKp44	O95944	
NCR3	259197	NKp30	O14931	
NGF	4803	b-NGF	P01138	
NID1	4811	Nidogen	P14543	
NID2	22795	NID2	Q14112	
NME1	4830	Nucleoside diphosphate kinase A	P15531	
NME2	4831	NDP kinase B	P22392	
NOG	9241	Noggin	Q13253	
NOV	4856	NovH	P48745	
NPPB	4879	BNP-32	P16860	
NR3C1	2908	Glucocorticoid receptor	P04150	
NRG1	3084	NEUREGULIN-1	Q02297	
NRP1	8829	NRP1	O14786	
NSFL1C	55968	NSF1C	Q9UNZ2	

Gene Name	GeneId	Protein Target	SwissProt ID	MM Biomarker Candidate
NTF3	4908	Neurotrophin-3	P20783	
NTF4	4909	Neurotrophin-5	P34130	
NTN4	59277	NET4	Q9HB63	
NTRK1	4914	TrkA	P04629	
NTRK2	4915	TrkB	Q16620	
NTRK3	4916	TrkC	Q16288	
NUDCD3	23386	NUDC3	Q8IVD9	
NXPH1	30010	NXPH1	P58417	
OCIAD1	54940	OCAD1	Q9NX40	
OLFM4	10562	Olfactomedin-4	Q6UX06	
OLR1	4973	OLR1	P78380	
OPCML	4978	OBCAM	Q14982	
OSM	5008	OSM	P13725	
OTUB1	55611	OTUB1	Q96FW1	
PA2G4	5036	PA2G4	Q9UQ80	
PAFAH1B2	5049	PAFAH beta subunit	P68402	
PAK3	5063	PAK3	O75914	
PAK6	56924	PAK6	Q9NQU5	
PAK7	57144	PAK7	Q9P286	
PAPPA	5069	PAPP-A	Q13219	
PCNA	5111	PCNA	P12004	
PCSK7	9159	PCSK7	Q16549	
PDCD1LG2	80380	PD-L2	Q9BQ51	
PDGFA	5154	PDGF-AA	P04085	
PDGFB	5155	PDGF-BB	P01127	
PDGFC	56034	PDGF-CC	Q9NRA1	
PDGFRB	5159	PDGF Rb	P09619	
PDIA3	2923	Protein disulfide isomerase A3	P30101	
PDPK1	5170	PDPK1	O15530	
PDXP	57026	PLPP	Q96GD0	
PEBP1	5037	prostatic binding protein	P30086	
PECAM1	5175	PECAM-1	P16284	
PES1	23481	PESC	O00541	
PEX5	5830	PEX5	P50542	
PF4	5196	PF-4	P02776	
PFDN5	5204	PFD5	Q99471	
PGAM1	5223	Phosphoglycerate mutase 1	P18669	
PGD	5226	6-Phosphogluconate dehydrogenase	P52209	
PGF	5228	PlGF	P49763	
PGK1	5230	phosphoglycerate kinase 1	P00558	
PGLYRP1	8993	PGRP-S	O75594	
PI3	5266	Elafin	P19957	
PIGR	5284	PIGR	P01833	
PIK3CA PIK3R1	5290 5295	PIK3CA/PIK3R1	P42336, P27986	

Gene Name	GeneId	Protein Target	SwissProt ID	MM Biomarker Candidate
PIK3CG	5294	PK3CG	P48736	
PKM2	5315	M2-PK	P14618	
PLA2G10	8399	GX	O15496	
PLA2G1B	5319	GIB	P04054	
PLA2G2A	5320	NPS-PLA2	P14555	
PLA2G2E	30814	GIIE	Q9NZK7	
PLA2G5	5322	GV	P39877	X
PLA2G7	7941	PAFAH	Q13093	
PLAT	5327	tPA	P00750	
PLAU	5328	uPA	P00749	
PLAUR	5329	suPAR	Q03405	
PLCG1	5335	PLCG1	P19174	
PLG	5340	Angiostatin	P00747	
PLG	5340	Plasmin	P00747	
PLG	5340	Plasminogen	P00747	
PLK1	5347	PLK-1	P53350	
PLXNC1	10154	PLXC1	O60486	
POMC	5443	ACTH	P01189	
POMC	5443	b-Endorphin	P01189	
PON1	5444	paraoxonase 1	P27169	
POR	5447	NADPH-P450 Oxidoreductase	P16435	
PPA1	5464	PPase	Q15181	
PPBP	5473	CTAP-III	P02775	
PPBP	5473	NAP-2	P02775	
PPBP	5473	NAP-2	P02775	
PPIA	5478	Cyclophilin A	P62937	
PPIB	5479	PPIB	P23284	
PPP1R1B	84152	DARPP-32	Q9UD71	
PPP3CA PPP3R1	5530 5534	Calcineurin	Q08209, P63098	
PPP3R1	5534	Calcineurin B a	P63098	
PPY	5539	PH	P01298	
PRDX1	5052	Peroxiredoxin-1	Q06830	
PRDX2	7001	Peroxiredoxin 2	P32119	
PRDX5	25824	Peroxiredoxin-5	P30044	
PRDX6	9588	Peroxiredoxin-6	P30041	
PRKACA	5566	PRKACA	P17612	
PRKCA	5578	PKC-A	P17252	
PRKCB	5579	PKC-B-II	P05771	
PRKCD	5580	PKC-D	Q05655	
PRKCG	5582	PKC-G	P05129	
PRKCI	5584	KPCI	P41743	
PRKCQ	5588	KPCT	Q04759	
PRKCZ	5590	PKC-Z	Q05513	
PRL	5617	PRL	P01236	
PROC	5624	Activated Protein C	P04070	

Gene Name	GeneId	Protein Target	SwissProt ID	MM Biomarker Candidate
PROC	5624	Activated Protein C	P04070	
PROC	5624	Protein C	P04070	
PROK1	84432	EG-VEGF	P58294	
PROS1	5627	Protein S	P07225	
PRSS1	5644	Trypsin	P07477	
PRSS2	5645	Trypsin 2	P07478	
PRSS22	64063	BSSP4	Q9GZN4	
PRSS27	83886	Marapsin	Q9BQR3	
PRSS3	5646	TRY3	P35030	
PRSS7	5651	Enterokinase	P98073	
PRTN3	5657	Proteinase-3	P24158	X
PSMA1	5682	PSA1	P25786	
PSMA2	5683	PSA2	P25787	
PSMA6	5687	PSA6	P60900	
PSMD7	5713	PSD7	P51665	
PSME1	5720	PSME1	Q06323	
PSPN	5623	Persephin	O60542	
PTEN	5728	pTEN	P60484	
PTGS2	5743	COX-2	P35354	
PTH	5741	PTH	P01270	
PTHLH	5744	PTHrP	P12272	
PTK2	5747	FAK1	Q05397	
PTK6	5753	PTK6	Q13882	
PTN	5764	PTN	P21246	
PTPN1	5770	PTP-1B	P18031	
PTPN11	5781	SHP-2	Q06124	
PTPN2	5771	TCPTP	P17706	
PTPN6	5777	PTP-1C	P29350	
PYY	5697	PYY	P10082	
RAC1	5879	RAC1	P63000	
RAD51	5888	RAD51	Q06609	
RAN	5901	RAN	P62826	
RARRES2	5919	TIG2	Q99969	
RASA1	5921	RASA1	P20936	
RB1	5925	Rb	P06400	
RBM39	9584	RBM39	Q14498	
RBP4	5950	RBP	P02753	X
RELT	84957	RELT	Q969Z4	
REN	5972	Renin	P00797	
RET	5979	RET	P07949	
RETN	56729	resistin	Q9HD89	
RGMA	56963	RGMA	Q96B86	
RGMB	285704	RGMB	Q6NW40	
ROR1	4919	ROR1	Q01973	
RPS27A	6233	Ubiquitin	P62988	

Gene Name	GeneId	Protein Target	SwissProt ID	MM Biomarker Candidate
RPS27A	6233	Ubiquitin+1	P62988	
RPS3	6188	RS3	P23396	
RPS3A	6189	RS3A	P61247	
RPS6KA3	6197	RPS6KA3	P51812	
RPS6KA5	9252	RSK-like protein kinase	O75582	
RPS7	6201	RS7	P62081	
RSPO2	340419	RSPO2	Q6UXX9	
RUNX2	860	Osteoblast-specif transcr fact 2	Q13950	
S100A12	6283	S100A12	P80511	
S100A7	6278	S100A7	P31151	
SAA1	6288	SAA	P02735	X
SBDS	51119	SBDS	Q9Y3A5	
SCT	6343	Secretin	P09683	
SELE	6401	sE-Selectin	P16581	
SELL	6402	sL-Selectin	P14151	
SELP	6403	P-Selectin	P16109	
SEMA3A	10371	Semaphorin 3A	Q14563	
SERPINA1	5265	a1-Antitrypsin	P01009	
SERPINA3	12	a1-Antichymotrypsin	P01011	
SERPINA4	5267	Kallistatin	P29622	X
SERPINA5	5104	PCI	P05154	
SERPINA6	866	CBG	P08185	
SERPINA7	6906	Thyroxine-Binding Globulin	P05543	
SERPINC1	462	Antithrombin III	P01008	
SERPIND1	3053	Heparin cofactor II	P05546	
SERPINE1	5054	PAI-1	P05121	
SERPINE2	5270	Protease nexin I	P07093	
SERPINF2	5345	a2-Antiplasmin	P08697	
SERPING1	710	C1-Esterase Inhibitor	P05155	
SETD7	80854	SET9	Q8WTS6	
SEZ6L2	26470	SE6L2	Q6UXD5	
SFRP1	6422	SARP-2	Q8N474	
SFTPD	6441	SP-D	P35247	
SGTA	6449	SGTA	O43765	
SH2D1A	4068	SH21A	O60880	
SHH	6469	Sonic Hedgehog	Q15465	
SIGLEC1	6614	Sialoadhesin	Q9BZZ2	
SIGLEC6	946	Siglec-6	O43699	
SIGLEC7	27036	Siglec-7	Q9Y286	
SIGLEC9	27180	Siglec-9	Q9Y336	
SIRT2	22933	SIRT2	Q8IXJ6	
SKP1	6500	SKP1	P63208	
SLITRK1	114798	SLIK1	Q96PX8	
SLITRK5	26050	SLIK5	O94991	
SLPI	6590	SLPI	P03973	

Gene Name	GeneId	Protein Target	SwissProt ID	MM Biomarker Candidate
SMPDL3A	10924	ASM3A	Q92484	
SNRPF	6636	RUXF	P62306	
SNX4	8723	Sorting nexin 4	O95219	
SOD1	6647	SOD	P00441	
SOD2	6648	Mn SOD	P04179	
SORCS2	57537	SORC2	Q96PQ0	
SPARC	6678	ON	P09486	
SPARCL1	8404	SPARCL1	Q14515	
SPHK1	8877	Sphingosine kinase 1	Q9NYA1	
SPHK2	56848	SPHK2	Q9NRA0	
SPINT1	6692	HAI-1	O43278	
SPINT2	10653	SPINT2	O43291	
SPOCK1	6695	Testican-1	Q08629	
SPOCK2	9806	Testican-2	Q92563	
SPON1	10418	Spondin-1	Q9HCB6	
SPTAN1	6709	SPTA2	Q13813	
SRC	6714	SRCN1	P12931	
SSRP1	6749	SSRP1	Q08945	
SST	6750	Somatostatin-28	P61278	
STAB2	55576	STAB2	Q8WWQ8	
STC1	6781	Stanniocalcin-1	P52823	
STIP1	10963	Stress-induced-phosphoprotein 1	P31948	
STK16	8576	STK16	O75716	
STX1A	6804	STX1a	Q16623	
SYNCRIP	10492	HNRPQ	O60506	
TACSTD2	4070	GA733-1 protein	P09758	
TBK1	29110	TBK1	Q9UHD2	
TBP	6908	TBP	P20226	
TDGF1	6997	Cripto	P13385	
TEC	7006	TEC	P42680	
TEK	7010	sTie-2	Q02763	
TF	7018	Transferrin	P02787	
TFF3	7033	TFF3	Q07654	
TFPI	7035	TFPI	P10646	
TG	7038	Thyroglobulin	P01266	
TGFB1	7040	TGF-b1	P01137	
TGFB2	7042	TGF-b2	P61812	X
TGFB3	7043	TGF-b3	P10600	
TGFBI	7045	BGH3	Q15582	
TGFBR3	7049	TGF-b R III	Q03167	
TGM3	7053	TGM3	Q08188	
THBS1	7057	Thrombospondin-1	P07996	
THBS2	7058	TSP2	P35442	
THBS4	7060	TSP4	P35443	

Gene Name	GeneId	Protein Target	SwissProt ID	MM Biomarker Candidate
THPO	7066	Tpo	P40225	
TIE1	7075	sTie-1	P35590	
TIMP1	7076	TIMP-1	P01033	X
TIMP2	7077	TIMP-2	P16035	
TIMP3	7078	TIMP-3	P35625	
TK1	7083	Thymidine kinase	P04183	
TKT	7086	Transketolase	P29401	
TLR2	7097	TLR2	O60603	
TLR4	7099	TLR4	O00206	
TNC	3371	Tenascin	P24821	
TNF	7124	TNF-a	P01375	
TNFAIP6	7130	TSG-6	P98066	
TNFRSF10A	8797	TRAIL R1	O00220	
TNFRSF10D	8793	TRAIL R4	Q9UBN6	
TNFRSF13B	23495	TACI	O14836	
TNFRSF13C	115650	BAFF Receptor	Q96RJ3	
TNFRSF17	608	BCMA	Q02223	
TNFRSF1A	7132	TNF sR-I	P19438	
TNFRSF1B	7133	TNF sR-II	P20333	
TNFRSF4	7293	TNR4	P43489	X
TNFRSF8	943	CD30	P28908	X
TNFRSF9	3604	4-1BB	Q07011	
TNFSF11	8600	sRANKL	O14788	
TNFSF12	8742	TWEAK	O43508	
TNFSF13	8741	APRIL	O75888	
TNFSF13B	10673	BAFF	Q9Y275	
TNFSF15	9966	TNFSF15	O95150	
TNFSF18	8995	TNFSF18	Q9UNG2	
TNFSF4	7292	OX40 Ligand	P23510	
TNFSF8	944	CD30 Ligand	P32971	
TNFSF9	8744	4-1BB ligand	P41273	
TNNI3	7137	Troponin I	P19429	
TOP1	7150	Topoisomerase I	P11387	
TPI1	7167	Triosephosphate isomerase	P60174	
TPM1	7168	Tropomyosin 1 alpha chain	P09493	
TPM2	7169	Tropomyosin 2	P07951	
TPO	7173	TMA	P07202	
TPSB2	64499	TPSB2	P20231	
TPSG1	25823	TPSG1	Q9NRR2	
TPT1	7178	TCTP	P13693	X
TSLP	85480	TSLP	Q969D9	
TXNDC12	51060	TXD12	O95881	
TYMS	7298	TS	P04818	
TYRO3	7301	Dtk	Q06418	
UBE2I	7329	UBC9	P63279	

Gene Name	GeneId	Protein Target	SwissProt ID	MM Biomarker Candidate
UBE2L3	7332	UB2L3	P68036	
UBE2N	7334	UBE2N	P61088	
UFC1	51506	UFC1	Q9Y3C8	
UFM1	51569	UFM1	P61960	
ULBP1	80329	ULBP-1	Q9BZM6	
ULBP2	80328	ULBP-2	Q9BZM5	
ULBP3	79465	ULBP-3	Q9BZM4	
VCAM1	7412	VCAM-1	P19320	
VEGFA	7422	VEGF	P15692	X
VEGFA	7422	VEGF121	P15692	
VEGFC	7424	VEGF-C	P49767	
VIP	7432	Vasoactive Intestinal Peptide	P01282	
VTA1	51534	DRG-1	Q9NP79	
VWF	7450	vWF	P04275	
WFDC2	10406	HE4	Q14508	
WFIKKN2	124857	WFKN2	Q8TEU8	
WIF1	11197	WIF-1	Q9Y5W5	
WISP1	8840	WISP-1	O95388	
WISP3	8838	WISP-3	O95389	
WNK3	65267	WNK3	Q9BYP7	
WNT7A	7476	WNT7A	O00755	
XCL1	6375	Lymphotactin	P47992	
XPNPEP1	7511	XPNPEP1	Q9NQW7	
XRCC6	2547	Ku70	P12956	
YES1	7525	YES	P07947	
YWHAG	7532	14-3-3 protein gamma	P61981	
YWHAH	7533	14-3-3 eta	Q04917	X
YWHAZ	7534	14-3-3 protein zeta/delta	P63104	
ZAP70	7535	ZAP70	P43403	
ZAP70	7535	ZAP70	P43403	
ZAP70	7535	ZAP70	P43403	
