# Supplementary figures and images for: Early Detection of Malignant Pleural Mesothelioma in Asbestos-Exposed Individuals with a Noninvasive Proteomics-Based Surveillance Tool
Source: PLoS One. 2012 Oct 3;7(10):e46091. doi: 10.1371/journal.pone.0046091 (PMC3463527; doi:10.1371/journal.pone.0046091)

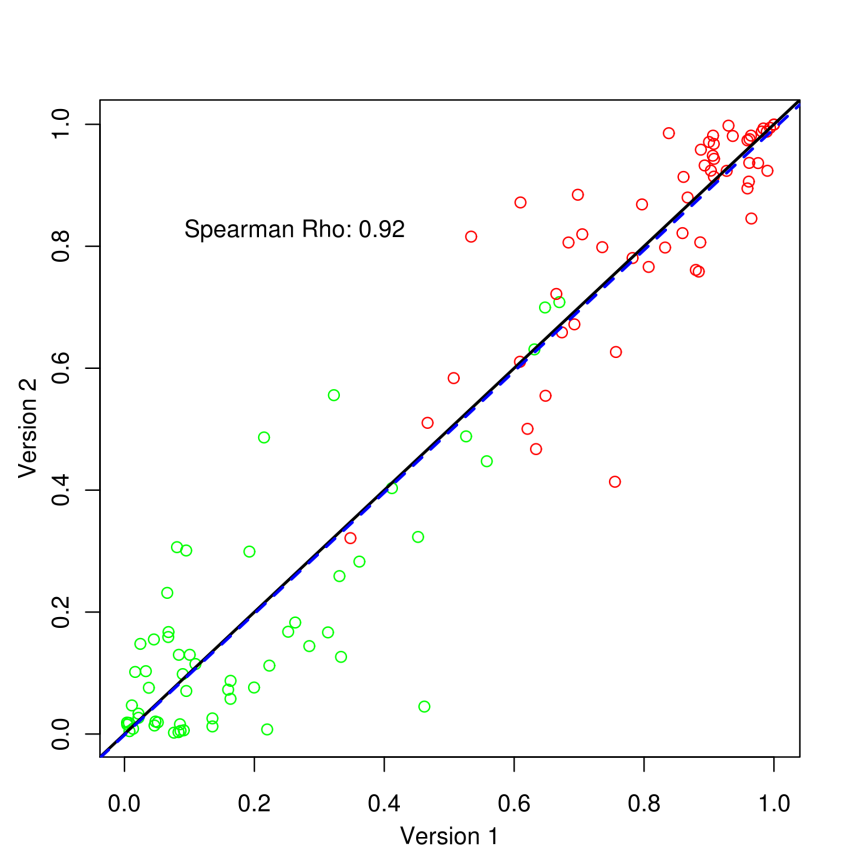

Supplement: Figure S1 — Plot of classifier prediction scores for the V1 and V2 classifier. The plot shows consistent predictions for both models on the same 113 samples present in both versions. MM samples are colored red and asbestos exposed patients are colored green. (DOCX) [file pone.0046091.s003.docx]
